# Mitotic SENP3 activation couples with cGAS signaling in tumor cells to stimulate anti-tumor immunity

**DOI:** 10.1038/s41419-022-05063-6

**Published:** 2022-07-22

**Authors:** Gaolei Hu, Yalan Chen, Xinyu Yang, Yang Wang, Jianli He, Tianshi Wang, Qiuju Fan, Liufu Deng, Jun Tu, Hongsheng Tan, Jinke Cheng

**Affiliations:** 1 Department of Biochemistry and Molecular Cell Biology, Shanghai Key Laboratory for Tumor Microenvironment and Inflammation, Shanghai Jiao Tong University School of Medicine, Shanghai, 200025 China; 2 School of Pharmacy, Shanghai Jiao Tong University, Shanghai, 200240 China; 3 Clinical Research Center, Shanghai Jiao Tong University School of Medicine, Shanghai, 200025 China

**Keywords:** Sumoylation, Cancer microenvironment

## Abstract

Our previous studies show that the mitotic phosphorylation of SUMO-specific protease 3 (SENP3) can inhibit its de-SUMOylation activity in G2/M phase of the cell cycle. Inhibition of SENP3 plays a critical role in the correct separation of sister chromatids in mitosis. The mutation of mitotic SENP3 phosphorylation causes chromosome instability and promotes tumorigenesis. In this study, we find that the mutation of mitotic SENP3 phosphorylation in tumor cells can suppress tumor growth in immune-competent mouse model. We further detect an increase of CD8^+^ T cell infiltration in the tumors, which is essential for the anti-tumor effect in immune-competent mouse model. Moreover, we find that mitotic SENP3 activation increases micronuclei formation, which can activate cGAS signaling-dependent innate immune response. We confirmed that cGAS signaling mediates the mitotic SENP3 activation-induced anti-tumor immunity. We further show that p53 responding to DNA damage activates mitotic SENP3 by inhibiting phosphorylation, and further increases cellular senescence as well as the related innate immune response in tumor cells. Furthermore, TCGA database demonstrates that the SENP3 expression positively correlates with the induction of innate immune response as well as the survival of the p53 mutant pancreatic cancer patients. Together, these data reveal that mitotic SENP3 activation in tumor cells can promote host anti-tumor immune response by coupling with cGAS signaling.

## Introduction

The main function of mitosis is to divide sister chromatids into two daughter cells. The correct segregation of chromatids is a key event to ensure the smooth division and proliferation of cells. The entire process of mitosis is finely regulated by protein modifications such as phosphorylation and ubiquitination [1]. External or internal factors dysregulate the process of mitosis to cause mis-separation of chromatids and induce chromosome instability, which is related to tumorigenesis [2–4]. On the other hand, the mis-segregation of chromatids often causes the accumulation of cytoplasmic DNA, mainly in the form of micronuclei (MN) [5–7].

MN as signal activates innate immune response via cGAS-STING signaling pathway, an evolutionarily conserved mechanism that initiates the host immune system to respond to microbial infections or cytosolic intrinsic self-DNA [8–10]. cGAS can detect cytosolic DNA and synthesize the second messenger cGAMP, which then binds STING and induces expression of type I interferon and other cytokines through IRF3/NF-κB signaling [11–14]. It is well known that antigen-presenting cells (APCs) can be activated by type I interferon signaling derived from tumor cells [15–17] and then present tumor antigens to CD8^+^ T cells [18, 19]. The cGAS–STING-mediated interferon production can also be induced by the direct interaction of APCs and tumor cells [20]. Interestingly, cancer cells have been shown to down-regulate cGAS and STING expression through hypermethylation of its promoter region in response to the antitumor functions of cGAS-STING signaling [16]. Moreover, MN derived from chromosomal instability is also reported to promote inflammation-driven metastasis [21, 22].

SUMO is a ubiquitin-like modifier and can covalently modify many proteins in diverse processes including regulation of transcription, chromatin structure, and signal transduction [23]. The whole process of SUMOylation is completed by three enzymes: activating enzymes (E1, SAE1/SAE2), conjugating enzymes (E2, UBE2I) and ligases (E3) [24]. As a reversible modification, SUMO can also be removed from the substrates by SENPs, a SUMO-specific protease with isopeptidase activity [25]. Recently, we and others find that SUMOylation also involves in the regulation of mitotic progression [26, 27]. SUMO-specific protease SENP3 is highly phosphorylated by CDK1 in G2/M phase of the cell cycle. Importantly, mutation of these phosphorylation sites can markedly enhance SENP3 de-SUMOylation activity, suggesting mitotic SENP3 phosphorylation as an inhibitory mechanism to control SENP3 activation in G2/M phase. Meanwhile, we show that the mutation of mitotic SENP3 phosphorylation causes chromosome instability and abnormal cell division [27], suggesting that turning off SENP3 activity is essential for maintaining chromosome stability in mitosis. We further identify that p53 activates SENP3 in G2/M phase to regulate DNA-damage-induced G2 arrest of the cell cycle [28]. These findings suggest that the regulation of mitotic SENP3 is a critical event to ensure chromosomes stability in G2/M phase of the cell cycle.

In this study, we show that mitotic SENP3 activation in tumor cells inhibits the tumor growth in immune-competent C57BL/6 mouse model, which is the opposite of what was observed in tumor-bearing nude mice model. We further find that activating mitotic SENP3 by mutating the phosphorylation sites increases the formation of micronuclei, which enhance cGAS signaling-dependent innate immune response in tumor cells followed by stimulating host CD8^+^ T cell-mediated anti-tumor immunity in C57BL/6 mice. In addition, p53 activates mitotic SENP3 to promote DNA damage-induced cellular senescence and innate immune response in tumor cells. TCGA database also demonstrates a positive correlation between SENP3 expression and inflammatory response as well as survival in p53 mutant pancreatic cancer patients. These results suggest that activating mitotic SENP3 in tumor cells can promote the host anti-tumor immunity.

## Results

### Activating mitotic SENP3 in tumor cells promotes host anti-tumor immunity

**Fig. 1:**
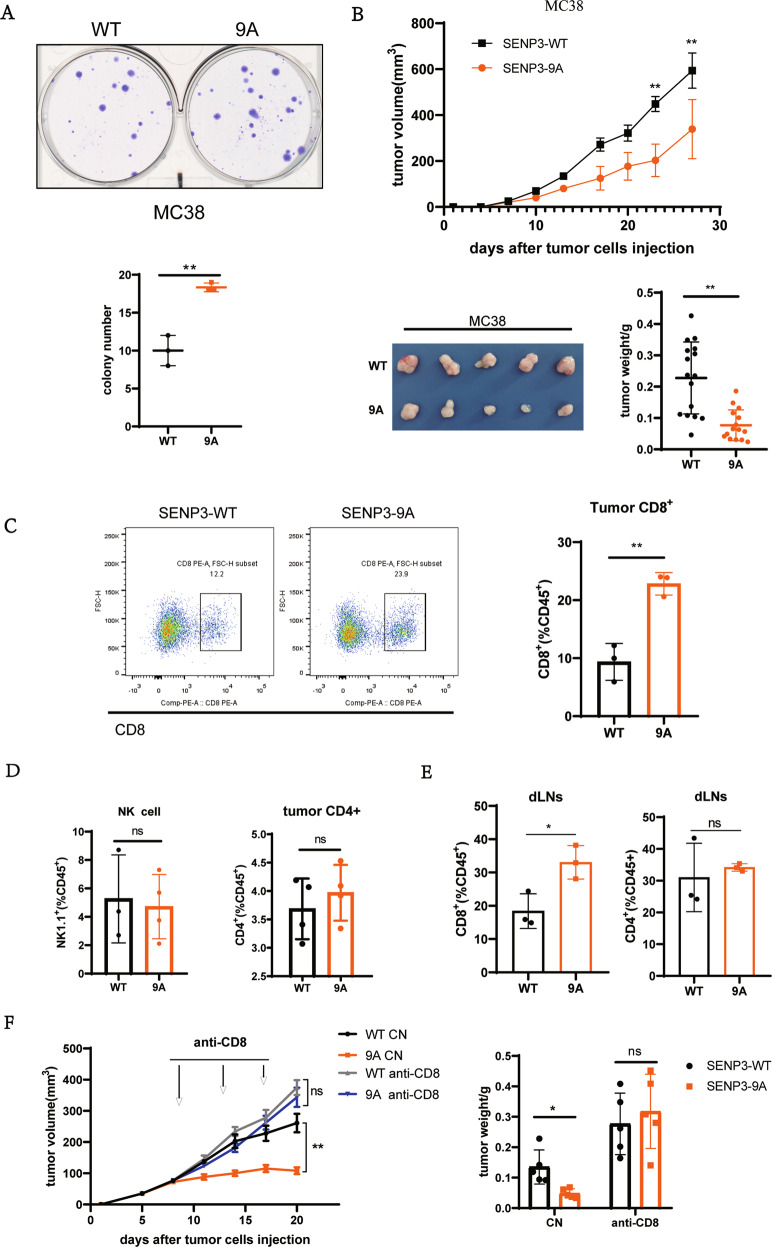
Activating mitotic SENP3 in tumor cells stimulates host anti-tumor immunity. **A** Colony formation assay in vitro. SENP3-WT-MC38 cells (WT) or SENP3–9A-MC38(9A) cells (500 cells/well) were cultured in six-well plate. Two weeks after seeding, the colonies containing more than 50 cells were recorded. Data are representative of three independent experiments and are shown as mean ± SD. **P < 0.01. **B** SENP3-WT-MC38 cells (WT) or SENP3–9A-MC38(9A) cells were subcutaneously injected into C57BL/6 mice (1 × 10^6^/mouse, *n* = 5 mice). Tumor size was determined once in 3 days in two dimensions. Tumor growth curves represent the mean ± SD. Tumors were harvested and pictured on day 28 post-injection. Tumor weight was also measured immediately after harvest and the data from three independent experiments were shown in the bottom panel. Mean ± SD is indicated. **P < 0.01. **C**, **D** SENP3-WT-MC38 cells (WT) or SENP3–9A-MC38(9A) cells were subcutaneously injected into C57BL/6 mice (1 × 10^6^/mouse, *n* = 5 mice). Tumors were harvested in 18 days’ post-injection and analyzed by flow cytometry (**C**). The cells were pre-gated on CD45^+^. Histogram shows the population of CD8^+^ T cells (**C**), NK1.1^+^, or CD4^+^ (**D**) in gated CD45^+^ cells. Data are representative of three independent experiments and are shown as mean ± SD. **P < 0.01. **E** SENP3-WT-MC38 cells (WT) or SENP3–9A-MC38(9A) cells were subcutaneously injected into C57BL/6 mice (1 × 10^6^/mouse, *n* = 5 mice). Tumor draining inguinal lymph nodes (dLNs) were removed from mice in 18 days’ post-injection. CD8^+^ or CD4^+^ T cells in CD45^+^ cells were analyzed by flow cytometry. Data are representative of three independent experiments and are shown as mean ± SD. *P < 0.05. **F** SENP3-WT-MC38 cells (WT) or SENP3–9A-MC38(9A) cells were subcutaneously injected into C57BL/6 mice (1 × 10^6^/mouse, *n* = 5 mice). On day 7 after injection, each kind of tumor-bearing mice was divided into two groups and anti-mouse CD8α antibodies were intraperitoneally injected (250 μg per mouse) on day 9, 13, and 17 after tumor injection. Tumor size was determined once in 3 days in two dimensions. Tumor growth curves represent the mean ± SD. Tumors were harvested on day 20 post-injection. Tumor weights were also measured immediately after harvest and the data were shown in histogram. Mean ± SD is indicated. **P < 0.01, *P < 0.05.

We have previously shown that activating mitotic SENP3 by mutating the nine phosphorylation sites causes chromosome instability and promotes tumorigenesis in in vitro or in tumor-bearing nude mice models [27]. We would like to further determine whether activating mitotic SENP3 in tumor cells could perform the same in immune-competent mice as in nude mice. To do so, we generated a mouse tumor cell line MC38 expressed with SENP3 wild-type (SENP3-WT) or mitotic SENP3 phosphorylation mutant (SENP3–9A, a mitotic activation form of SENP3). The colony formation assay showed that SENP3–9A-MC38 cells grown more colonies than SENP3-WT-MC38 did (Fig. 1A), a consistent phenotype shown in human tumor cell U2OS [27]. However, we generated MC38 cell subcutaneous tumor model in C57BL/6 mice and showed that the SENP3–9A MC38 tumors grown much smaller than the SENP3-WT MC38 tumors in C57BL/6 mice (Fig. 1B). Obviously, this result was opposite to what we observed previously in tumor-bearing nude mice model [27].

Considering SENP3–9A tumor growing in immune-competent mice, we reasoned that SENP3–9A in tumor cells could induce host anti-tumor immunity to inhibit tumor growth in immune-competent mice. To test it, we performed FACS assay in CD45^+^ cells from tumors or from draining lymph nodes near the tumors. The population of CD8^+^ T cells but not NK cells or CD4^+^ T cells in CD45^+^ cells from the SENP3–9A tumor tissues were much more than that from the SENP3-WT tumors in C57BL/6 mice (Fig. 1C, D). A similar analysis of CD45^+^ immune cells from the draining lymph nodes near the tumor also showed that the population of CD8^+^ T cells but not CD4^+^ T cells were much more in the SENP3–9A tumor model than in the SENP3-WT tumor model (Figs. 1E and S1B). These data suggest that SENP3–9A expression in tumor cells can promote CD8^+^ T cell infiltration into tumors. To further determine the role of CD8^+^ T cells in the SENP3–9A-induced anti-tumor activity, we eliminated CD8^+^ T cells by injection of anti-CD8 antibody via intra-peritoneal in these tumor-bearing mice. We observed that anti-CD8 antibody treatment not only eliminated the difference in tumor growth between SENP3-WT and SENP3–9A MC38 tumor mice, but also increased tumor growth in both tumor models as compared to non-injected controls (Figs. 1F and S1C), suggesting that activating mitotic SENP3 in tumors can promote host CD8^+^ T cell-mediated anti-tumor immunity.

### Activating mitotic SENP3 enhances cGAS signaling

**Fig. 2:**
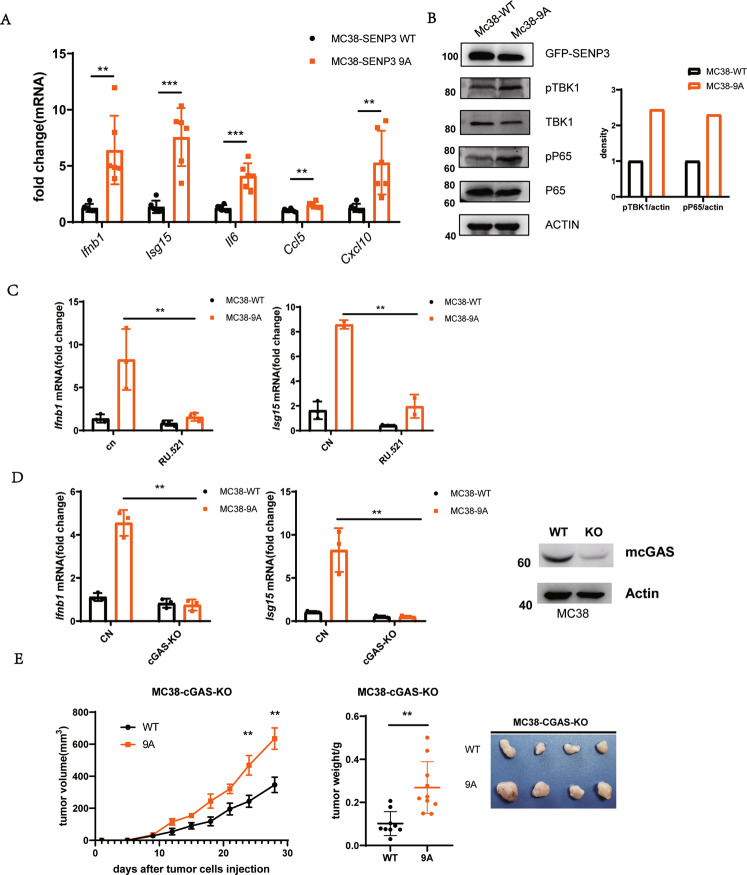
Mitotic SENP3 activates cGAS signaling. **A** Real-time PCR was used for the analysis of *Ifnb1, Isg15, Il6, Ccl5*, and *Cxcl10* expression in SENP3-WT-MC38 cells (WT) or SENP3–9A-MC38(9A) cells. The data were normalized to *Gapdh* internal control and represented as mean with SD (*n* = 3 independent biological replicates). **p < 0.01, ***p < 0.001. **B** Cell lysates from SENP3-WT-MC38 cells (WT) or SENP3–9A-MC38(9A) cells were blotted with anti-SENP3, anti-phosph-TBK1(pTPK1), anti-TBK1, anti-phosph-P65(pP65), anti-P65, or anti-actin antibodies. **C** Real-time PCR was used for analysis of *Ifnb1* and *Isg15* expression in SENP3-WT-MC38 cells (WT) or SENP3–9A-MC38(9A) cells treated with or without RU.521 for 2 days. The data were normalized to *Gapdh* internal control and represented as mean with SD (*n* = 3 independent biological replicates). **p < 0.01. **D** cGAS in SENP3-WT-MC38 cells (WT) or SENP3–9A-MC38(9A) cells was knocked out by using Cas9/CRISPER system plasmid px459 encoding cGAS gRNAs (left panel). Real-time PCR was used for the analysis of *Ifnb1* and *Isg15* expression in these cells. The data were normalized to *Gapdh* internal control and represented as mean with SD (*n* = 3 independent biological replicates). **p < 0.01. **E** C57BL/6 mice were subcutaneously injected with cGAS-knockout SENP3-WT-MC38 cells (WT) or SENP3–9A-MC38(9A) cells (1 × 10^6^/mouse). Tumor size was determined once in 3 days in two dimensions. Tumor growth curves represent the mean ± SD. Tumors were harvested and pictured on day 28 post-injection. Tumor weight was also measured immediately after harvest and the data from three independent experiments were shown in the bottom panel. Mean ± SD is indicated. **P < 0.01.

We next addressed how mitotic SENP3 activation in tumor cells promotes host anti-tumor immunity. We hypothesized that mitotic SENP3 activation would enhance the innate immune response in tumor cells to induce host anti-tumor immunity. To test so, we first compared the expression of innate immunity-related genes in SENP3-WT- and SENP3–9A-MC38 cells. As shown in Fig. 2A, type I interferon gene (*Ifnb1)* and some other innate immunity-related genes such as *Isg15, Il6, Ccl5*, and *Cxcl10* expressed much higher in SENP3–9A-MC38 cells than in SENP3-WT-MC38 cells. Meanwhile, we observed a similar effect of SENP3–9A on innate immune response in human cancer cell line U2OS cells. We detected a higher secreted amount of IL-6 or IL-8 in the culture medium of SENP3–9A-U2OS cells than that in the culture medium of SENP3-WT- U2OS cells (Fig. S2A). Furthermore, we also detected a higher expression level of *IFNB1, CCL5* in SENP3–9A-U2OS cells as compared to SENP3-WT- U2OS cells (Figs. S2C and 4C). The expression of SENP3–9E, a phosphorylation mimic mutant, showed almost the same effect on the expression of these genes as SENP3-WT did (Fig. S2C). However, knockdown of SENP3 expression did not affect *IFNB1* expression but reduced *CCL5* in U2OS cells (Fig. S2D). These results suggest that mitotic SENP3 activation can modulate the innate immune response in tumor cells.

Since mitotic SENP3 activation is related to DNA damage response, and cGAS-STING signaling is a critical pathway to bridge DNA damage to host antitumor immune responses [29], we thus reasoned that mitotic SENP3 may activate cGAS-STING signaling leading to the involvement of host immune components. Indeed, we compared the activation of cGAS-STING signaling in both SENP3-WT- and SENP3–9A tumor cells. The phosphorylation of TBK1, p65, or IRF3, as indicators of cGAS-STING signaling, was demonstrated higher in both SENP3–9A-MC38 cells and SENP3–9A-U2OS cells than in their SENP3-WT- control cells (Figs. 2B, S2B). We found that the cGAS inhibitor RU.521 markedly reduced the expression of *Ifnb1* and *Isg15* mRNA in SENP3–9A-MC38 cells, although the reduction was also slightly occurred in SENP3-WT cells (Fig. 2C). In addition, we used cGAS gRNA to reduce cGAS expression in MC38 cells and observed a decrease of expression of *Ifnb1* and *Isg15* in SENP3–9A-MC38 cells with cGAS knockdown. These data suggest an essential role of cGAS in SENP3–9A-induced innate immune responses (Fig. 2D). Meanwhile, we also observed that the reduction of cGAS expression reversed SENP3–9A-mediated inhibition of tumor growth in C57BL/6 mice (Fig. 2E). Together, these data show that mitotic SENP3 activates cGAS signaling-mediated innate immune response in tumor cells.

### Activating mitotic SENP3 promotes micronucleus formation

**Fig. 3:**
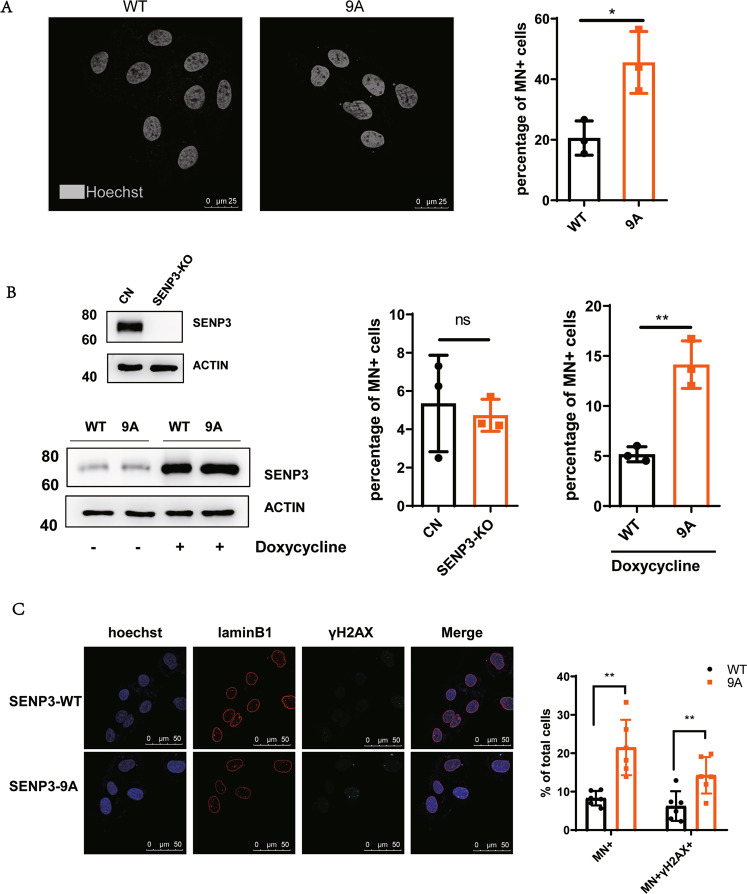
Mitotic SENP3 activation promotes micronucleus formation. **A** SENP3-WT- or SENP3–9A-U2OS cells were stained with Hoechst. The micronuclei were analyzed by using a confocal microscope. The micronucleus staining positive cells (percentage of total cells) were quantitatively analyzed on 30 fields pictured by a confocal microscope. Data are represented as mean with SD. *P < 0.05. **B** SENP3 in U2OS cells were knocked out by using Cas9/CRISPER system plasmid px459 encoding human SENP3 gRNAs (Top of left panel). Then SENP3 KO U2OS cells were stably transfected with Doxycycline-induced SENP3-WT (WT) or SENP3 9A (9A) plasmids. The expression of SENP3-WT or SENP3–9A mutant in these cells were induced by the addition of doxycycline(1 μM/mL) for 48 h (bottom of left panel). The micronuclei were stained with Hoechst and quantitatively analyzed on 30 fields pictured by confocal microscope (right panel). Data are represented as mean with SD. **P < 0.01. **C** SENP3-WT- or SENP3–9A-U2OS cells were immune-stained with anti-Lamin B1 or anti-γH2AX antibody, and co-stained with DAPI. The co-localization of micronuclei with γH2AX was quantitatively analyzed on 30 fields pictured by confocal microscope. Data are represented as mean with SD. **P < 0.01.

Cancer cells harbor mitotic chromosomal instability, which often forms micronuclei or cytoplasmic chromatin fragments [30]. Since activating mitotic SENP3 by de-phosphorylation increases chromosome instability in mitosis [27], we thus reasoned that activating mitotic SENP3 would promote the formation of micronuclei to activate cGAS signaling. Indeed, we stained U2OS cells with Hoechst and observed much more Hoechst positive foci in the cytoplasm of SENP3–9A U2OS cells than that in the cytoplasm of SENP3-WT cells. Quantitative analysis demonstrated that the micronucleus positive cells were up to ~50% among SENP3–9A-U2OS cells as compared to ~20% positive cells among SENP3-WT-U2OS cells, confirming that SENP3–9A expression increases the formation of micronuclei in tumor cells (Fig. 3A). Moreover, we generated a doxycycline-inducible SENP3 expression system in SENP3-knockout U2OS cells. Although doxycycline induced the same level of SENP3-WT or SENP3–9A proteins in SENP3-KO cells, SENP3–9A expression markedly increased the population of micronucleus positive cells from ~5% to ~15%, while SENP3-WT expression almost had no effect on micronucleus formation in SENP3-knockout U2OS cells (Fig. 3B). In addition, SENP3 phosphorylation mimics mutant SENP3–9E showed the similar effect on micronucleus formation as SENP3-WT did in U2OS cells (Fig. S3A and B), suggesting that the inactivation of mitotic SENP3 can reduce the micronucleus formation. As DNA damage is often involved in micronucleus formation [31–33], we stained γH2AX and showed that the γH2AX positive presented in the most of micronuclei in both cells (Fig. 2C). These results suggest that mitotic SENP3 activation causes DNA instability to increase micronucleus formation, which provides a niche for DNA recognition by cGAS in tumor cells.

### P53 activates mitotic SENP3 to promote innate immune response

**Fig. 4:**
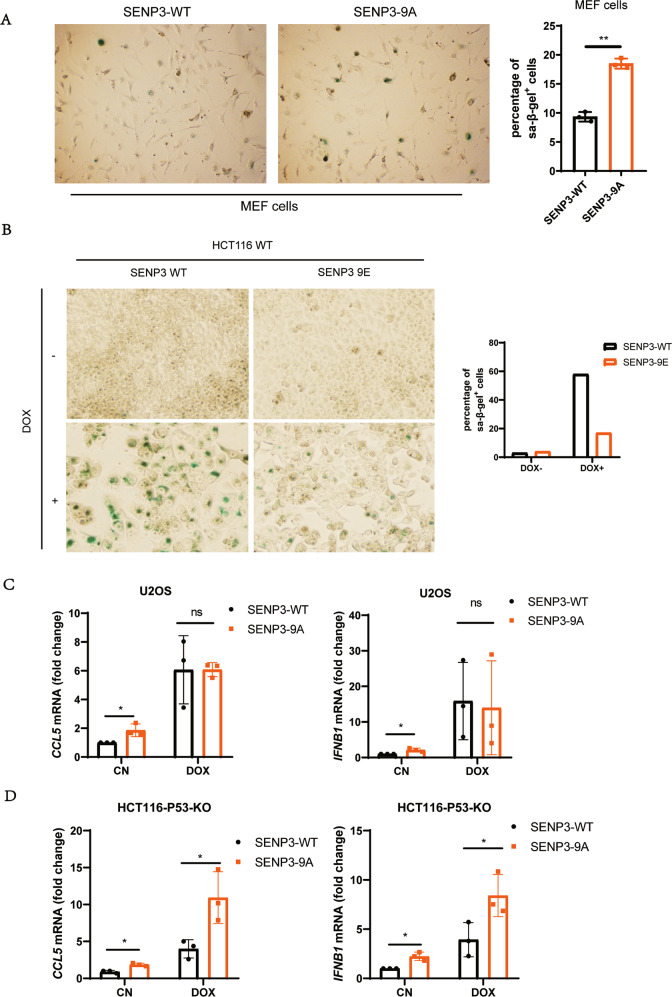
Mitotic SENP3 activation promotes cell senescence. **A** MEF cells were stably transfected with mouse SENP3-WT or SENP3–9A plasmids. Then these cells were stained with SA-β-Gal (left panel). SA-β-Gal positive cells were quantitatively analyzed on 30 fields pictured by microscope (right panel). Data are represented as mean with SD. **P < 0.01. **B** p53-WT HCT116 cells were stably transfected with SENP3-WT or SENP3–9E plasmids and then treated with doxorubicin (1 μg/ml) for 1 h. Then these cells were stained with SA-β-Gal in 6 days after doxorubicin treatment. **C** SENP3-WT- or SENP3–9A-U2OS cells were treated with or without doxorubicin (1 μg/ml) for 1 h and harvested in 3 days after treatment. Real-time PCR was used for the analysis of *CCL5* and *IFNB1* expression in these cells. The data were normalized to *GAPDH* internal control and represented as mean with SD (*n* = 3 independent biological replicates). *P < 0.05. (ns) No significant. **D** p53-KO HCT116 cells were stably transfected with SENP3-WT or SENP3–9A plasmids. These cells were treated with or without doxorubicin (1 μg/ml) for 1 h and harvested 3 days after treatment. Real-time PCR was used for the analysis of *CCL5* and *IFNB1* expression in these cells. The data were normalized to *GAPDH* internal control and represented as mean with SD (*n* = 3 independent biological replicates). *P < 0.05. (ns) No significant.

Our previous studies have shown that cell cycle negative regulator p53/p21 can inhibit mitotic SENP3 phosphorylation to turn on SENP3 deSUMOylation [28]. It is known that p53/p21 signaling can promote cellular senescence [34–37], which can activate cGAS-STING signaling [29, 38] and innate immune responding to DNA damage [39]. We thus reasoned that mitotic SENP3 may involve in DNA damage/p53-induced cell senescence and the related innate immunity. Indeed, we observed that SENP3–9A expression increased much more SA-β-gal staining positive cells in MEF cells than SENP3-WT did (Fig. 4A). Meanwhile, we found that SENP3 phosphorylation mimics mutant SENP3–9E markedly reduced doxorubicin-induced cellular senescence in p53 WT-HCT116 cells (Fig. 4B). These data suggest a critical role of mitotic SENP3 activation in DNA damage/p53-induced cellular senescence. As senescence cells can secret inflammatory cytokines or factors, we then reasoned that mitotic SENP3 would modulate the cellular senescence-mediated innate immune response. To do so, we analyzed *IFNB1* and *CCL5*, representative cytokines as senescence-associated secretory phenotypes [9, 10, 29]. As expected, doxorubicin treatment induced the expression of *IFNB1* and *CCL5* in U2OS cells. Like doxorubicin treatment, SENP3–9A expression also induced the expression of *IFNB1* and *CCL5* in U2OS cells. Interestingly, we found that SENP3-WT induced much less expression of *IFNB1* and *CCL5* than SENP3–9A did in p53-KO HCT116 cells under doxorubicin treatment (Fig. 4D). However, there were no difference in the expression of *IFNB1* and *CCL5* between SENP3-WT- and SENP3–9A-transfected U2OS cells (wild type for p53) under doxorubicin treatment (Fig. 4C). These data indicate that p53 inhibition of mitotic SENP3 phosphorylation plays a critical role in DNA damage-induced innate immune response.

### Positive correlation between SENP3 and immune response in p53 mutant pancreatic cancer patients

**Fig. 5:**
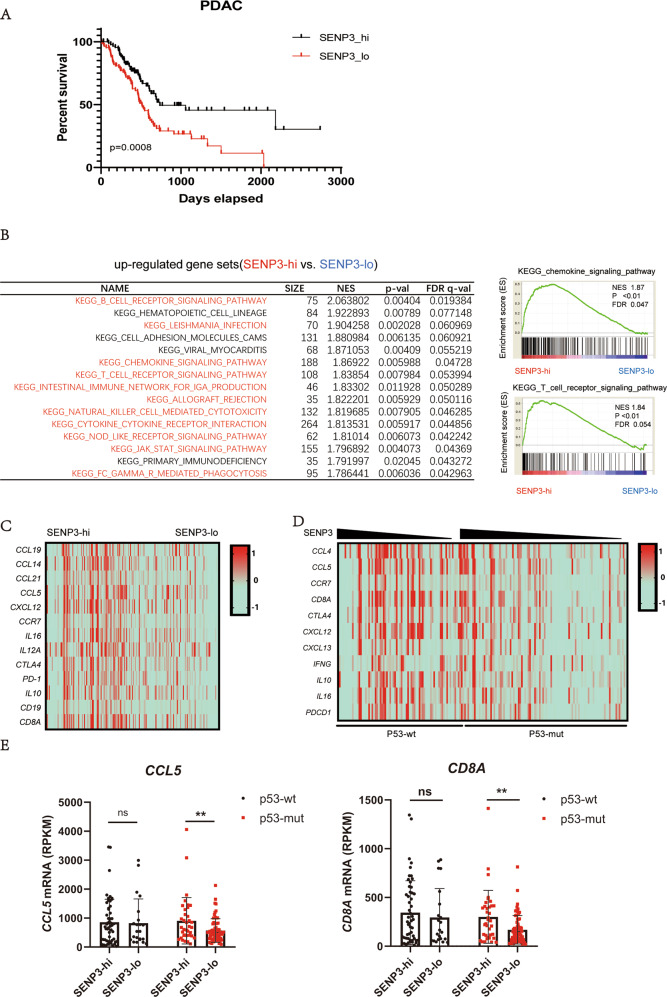
SENP3 expression positively correlated with immune response in p53 mutant pancreatic cancer patients [40]. **A** Kaplan–Meier curves of human PDAC patients with SENP3 high or low. **B** Gene Sets Enrichment Assay (GSEA) is used to analyze RNA sequence data of PDAC patients from TCGA database. The list shows the top pathways upregulated in SENP3 high vs. SENP3 low and shows that chemokine signaling and TCR signaling-related genes were enriched in SENP3 high patients. **C** Heatmaps show that immune response-related genes are positively related to SENP3 expression level in PDAC patients from TCGA database. **D** Heatmaps show that immune response-related genes are a positive correlation with SENP3 expression levels in p53 mutant PDAC patients from TCGA database. **E** The high mRNA level of *CCL5* and *CD8A* is shown in SENP3 high PDAC patients with p53 mutant but not the patients with p53 wild type.

Since p53 activates mitotic SENP3 to promote innate immune, we would like to explore the relationship among p53, SENP3, and innate immune response in tumor samples. We used TCGA data from the elapsed pancreatic cancer patients for the analysis. The elapsed pancreatic cancer samples from TCGA data [40] show that SENP3 expression is a positive correlation factor with the survival time of patients (Fig. 5A). We thus chose the pancreatic cancer patients with chemotherapy [40] to further analyze the relationship between p53, SENP3 expression, and the immune response. These patients were grouped as p53 wild-type or p53 mutant based on their genomic DNA sequence data. These patients were also grouped as SENP3 high or SENP3 low based on their SENP3 mRNA level in those tumor tissues. Due to mitotic SENP3 activation in tumor cells play a critical role in regulation of host anti-tumor immunity, we assume the high SENP3 expression may represent high SENP3 activity including in mitosis of tumor cells in these patients (Fig. S4C). Interestingly, the Gene Sets Enrichment Assay (GSEA) [41] shows that most of top upregulated gene sets in SENP3 high vs. SENP3 low are related to inflammation and immune response, which are enriched in SENP3 high tumor samples from the pancreatic cancer patients with chemotherapy (Fig. 5B, C). We further analyzed the effect of p53 and SENP3 on the expression of immune response-related genes. Generally, the expression of these immune response-related genes is mildly higher in p53 WT tumor samples as compared to that in p53 mutant samples (Fig. S4A). However, SENP3 expression shows a markedly positive correlation with the expression of these genes in tumors from p53 mutant patients, although a slight correlation also existed in the p53 WT tumor samples (Fig. 5D, E). In addition, SENP3 expression is positively correlated with the survival time of p53 mutant patients but not p53 wild-type patients (Fig. S4B). Put together, these data suggest that SENP3 is a positive regulator for immune response in p53 mutant pancreatic cancer patients.

## Discussion

Our previous studies identify SENP3 as a phosphorylated protein at G2/M phase of the cell cycle [27]. CDK1 phosphorylates SENP3 to turn off its de-SUMOylation activity at G2/M phase [27], which is essential for chromosome stability in mitotic program [26, 42]. The phosphorylation mutation can activate mitotic SENP3 and lead to chromosome instability and tumorigenesis [27]. Under DNA damage, p53 signaling activates SENP3 by reducing mitotic SENP3 phosphorylation and leads to G2 arrest of cell cycle [28]. In this study, we demonstrate that mitotic SENP3 activation can promote innate immune response in tumor cells, which induces host anti-tumor immunity to suppress tumor growth.

Although we have no direct evidence to support for the micronuclei as essential for mitotic SENP3-activated innate immune response, we identify cGAS as an essential regulator for the mitotic SENP3-activated innate immune response in tumor cells via promoting micronucleus formation. We have shown that mitotic SENP3 activation increases abnormal mitosis-caused chromatin instability [27], which leads to nucleus DNA strands releasing into the cytoplasm and the formation of micronucleus in tumor cells to activate cGAS signaling. The cGAS-STING signaling can sense nucleus DNA in cytoplasm and micronuclei to activate innate immunity [9, 20, 43]. Chemotherapy or radiotherapy causes genomic DNA damage, which often induces the secretion of inflammatory cytokines via cGAS-STING signaling [44]. These inflammatory cytokines can induce the enrichment of immune cells in the tumor microenvironment and increases anti-tumor immunity [43]. In the absence of cGAS, chemotherapy-induced innate immune response and anti-tumor effects are significantly reduced [45, 46]. Chemotherapy has been shown to promote the formation of micronucleus [30, 47]. The fragments of DNA in the micronucleus can be recognized by cGAS when its nuclear membrane ruptures [10]. It is conceivable that mitotic SENP3 activation would be a critical target for chemotherapy-induced anti-tumor immunity.

Mitotic SENP3 activation has an obvious anti-tumor effect in the immune-competent mouse models. The anti-CD8 antibody can deplete mitotic SENP3 activation-mediated anti-tumor effect, suggesting that CD8^+^ T cells play a critical role in mitotic SENP3 activation-associated anti-tumor immunity. We also observed that anti-CD8 antibodies can increase tumor growth in SENP3-WT tumors, suggesting that such CD8^+^ T cells may have broad anti-tumor effects but not only response for SENP3–9A-mediated anti-tumor immunity.

Chemotherapeutic drugs, as p53 inducer, can activate mitotic SENP3 by reducing the phosphorylation [28, 48]. Since mitotic SENP3 activation can induce innate immune response, chemotherapy can promote innate immune response via p53-mitotic SENP3 activation. We analyze the relationship between SENP3 expression level and tumor patient prognosis. Interestingly, we find a significant correlation between SENP3 expression level and prognosis in pancreatic cancer patients but not in other tumors like non-small cell lung cancer and triple-negative breast cancer patients (data not shown). Moreover, we further find that SENP3 expression positively correlates with the expression of immune response genes in p53 mutant pancreatic cancer patients from TCGA database, suggesting that increasing SENP3 expression can be instead of reducing mitotic SENP3 phosphorylation to activate innate immune response even in p53-mutant tumors. The findings further show that SENP3 is a positive factor to regulate anti-tumor immunity in tumor patients.

### Limitations of the study

In this study, we showed that the mitotic SENP3 phosphorylation modulates host anti-tumor immunity by regulating MN-induced cGAS-STING signaling. We did these observations by using SENP3 phosphorylation mutant tumor cell line in vitro or tumor mice model. The limitation is that we did not validate these results in tumor patients, as without suitable anti-SENP3 phosphorylation antibodies for immunostaining. If we have such anti-SENP3 phosphorylation antibody suitable for immunostaining patient tumor samples, we can screen mitotic SENP3 phosphorylation status in patient tumor samples and determine whether it is related to anti-tumor immunity in patients. If mitotic SENP3 phosphorylation can be used as a marker of anti-tumor immunity, it will be applied as an indicator to determine suitable patients for using anti-tumor immunotherapy.

## Methods

### Cell lines and reagents

MC38, U2OS, and HCT116 cell lines were cultured in DMED medium (Gibco) with 10% FBS, 1% PS at 37 °C, and 5% CO_2_. *SENP3*^−/−^ U2OS and *Cgas*^−/−^ MC38 cell lines were generated with CRISPER/cas9 technology [49]. *SENP3* forward 5′-CACCGAGCAGGTTTTTCGATGAGT-3′; *SENP3*-revers 5′-AAACACTCATCGAAAA ACCTGCTC-3′. *Cgas* sequence was referred to report of Zhijian J. Chen’s lab [50]. To establish SENP3 stably transfected cells, plasmids pCDH-SENP3, psPAX2, pMD2G were transfected into 293T cells to produce retrovirus, then these viruses were used to infect MC38 or U2OS cells. Stable cell lines were selected using 2 μM puromycin (Yeasen) for over 3 days. For cGAS inhibition, MC38 cells were treated with RU.521 (2 μg/mL) for two days before harvest. For DNA damage induction, U2OS cells or HCT116 cells were treated with doxorubicin (1 μg/mL) for 1 h and then washed with PBS two times before adding fresh DMEM medium (with 10% FBS,1% PS).

### Tumor model and flow cytometry

SENP3-WT- or SENP3–9A-MC38 cells (1 × 10^6^/mice) were inoculated into C57BL/6 mice subcutaneously. After 2–3 weeks, mice were dissected and the tumors were cut into appropriate sizes. Single-cell suspensions were generated by grinding and filtering. For flow cytometry assay, the collected cells were suspended in cell staining buffer (Biolegend, 420201), and then counted using the Countess II automated cell counter. 4 × 10^6^ cells from each group were used for flow cytometry staining. Stained cells were detected by the BD FACS Verse cell flow cytometer. Antibodies including APC-CD4 (100412), PE-CD8 (100708), Pecy5.5-CD45 (368504) were purchased from Biolegend. NK1.1-PE (12-5941-82) were purchased from ebioscience. Age and sex-matched male and female adult (6–8 week old) mice were used in each independent experiment. The animal experiments were performed in strict accordance with the “Guide for the Care and Use of Laboratory Animals”, which was approved by the Experimental Animal Ethical Committee at Shanghai Jiao Tong University School of Medicine.

### CD8^+^ T cells elimination in mouse tumor model

The anti-mouse CD8α antibody (BE0061) was purchased from BioXcell. Anti-mouse CD8α antibody (250 μg/mice) was used to inject intraperitoneally into tumor-bearing mice on day 9, 13, or 17 after tumor cell injection.

### Colony formation

MC38 cells were trypsinized and counted with a hemocytometer. The cells were diluted to 500 cells/2 ml using DMEM full medium and then plated in each well of the six-well plates. The cells were maintained at 37 °C to allow colony formation. Fourteen days later, the cells were fixed with PBS with 4% paraformaldehyde and stained with 0.5% crystal violet (Sigma-Aldrich). Colonies per well were counted and numbers were recorded from three independent experiments.

### Western blot analysis

Whole-cell extracts were prepared by lysis and sonication of cells in RIPA buffer (50 mM TRIS, 150 mM NaCl, 0.1% SDS, 1% NP40, 0.5% Sodium deoxycholate, 2 mM EDTA) and then analyzed using standard SDS-PAGE procedures. The primary antibodies were incubated in TBST buffer with 5% BSA over night at 4 °C. The second antibodies were incubated in TBST buffer for 1 h at room temperature. Proteins were visualized with the immobilon western chemilum HRP substrate (Millipore) and imaged by using the Chemiluminescence Imaging System. Antibodies p65 (Cat# 4764, RRID: AB_823578), pP65 (Cat# 3033), TBK1 (Cat# 3504, RRID: AB_2255663), P-TBK1 (Cat# 5483 S), cGAS (Cat# 15102) were purchased from Cell signaling technology. IRF3 (Cat# Ab25950), p-IRF3 (Cat# Ab138449) were purchased from Abcam. Actin was purchased from Proteintech.

### Immunofluorescence staining

Cells grown on glass coverslips were washed and fixed in PBS with 4% paraformaldehyde (PFA) for 15 min at room temperature. After washing, cells were permeabilized with PBS containing 0.25% Triton X-100 plus 1% BSA at 4 °C for 10 min. The cells were blocked in TBST buffer containing 1% BSA for more than 1 h. Primary antibodies were diluted with TBST buffer containing 1% BSA and the coverslips were incubated at room temperature for 1 h. The second antibodies were incubated for 1 h at room temperature. Coverslips were mounted using Antifade Mounting Medium with DAPI (ThermoFisher Scientific) and imaged by using a Leica TCS SP8 STED confocal microscopy. Antibodies γH2AX (Cat# ab26350) were purchased from Abcam, Lamin B1 (Cat# 12987-1-AP, RRID: AB_2136290) was purchased from Proteintech.

### SA β-gal staining

SA β-gal staining kit was purchased from Beyotime. Based on the manufacturer's instructions, cells were cultured in 6 cm dish, and fixed with 2 ml fix solution (Beyotime) for 15 min at room temperature followed by staining with 2 mL staining solution (Beytotime) at 37 °C (without CO_2_) over night. The 6 cm dish was pictured, and the stained positive cells were counted.

### qRT–PCR

Total cellular RNA was isolated by using TRIzol reagent according to the manufacturer’s instructions (Tiangen). cDNA was synthesized from the purified RNA by using fastking gDNA dispelling RT supermix (Tiangen). qPCR was performed using a SYBR Green Supermix PCR kit (Roche). Real-time qRT-PCR was performed using gene-specific primer sets (Supplementary Table 1). Gene expression was assessed in triplicate and normalized to a reference gene, GAPDH.

### Statistical analysis

Statistical analysis was performed by two-tailed Student’s *t* tests. The results of TCGA were based upon data generated by the TCGA Research Network (http://cancergenome.nih.gov RRID:SCR_003193). And the RNA-seq data sets were obtained from cBioportal (http://www.cbioportal.org). For a given cancer type (pancreatic cancer), tumor samples were ranked based on targeted gene (SENP3) expression values and were evenly divided into two groups accordingly. Statistical comparisons were then performed between the two groups for inflammatory genes or the housekeeping gene (GAPDH), as denoted.

**Key Resources Table**

**Table:** 

Reagent or resource	Source	Identifier
*Antibodies*		
Purified anti-mouse CD16/32	Biolgend	Cat# 101302;
APC anti-mouse CD4	Biolgend	Cat# 100412
PE anti-mouse CD8	Biolgend	Cat# 100708
Anti-mouse β-Actin (Actin)	Abcam	Cat# ab8227
Pecy5.5 anti-mouse CD45	Biolgend	Cat# 368504
PE anti-mouse NK1.1	ebioscience	Cat# 12-5941-82
anti-mouse CD8α antibody	BioXcell	Cat# BE0061
Anti-goat Lamin B	Santa Cruz	Cat# sc-6216; RRID: AB_648156
Anti-P65	CST	Cat# 4764, RRID:AB_823578
Anti-pP65	CST	Cat# 3033
Anti-TBK1	CST	Cat# 3504, RRID: AB_2255663
Anti-pTBK1	CST	Cat# 5483 S
Anti-cGAS	CST	Cat# 15102
Anti-IRF3	Abcam	Cat# Ab25950
Anti-pIRF3	Abcam	Cat# Ab138449
Anti-rabbit IgG HRP-linked	CST	Cat# 7074; RRID: AB_2099233
Anti-mouse IgG HRP-linked	CST	Cat# 7076; RRID: AB_330924
Complete protease inhibitor cocktail tablets	Roche	Cat#11697498001
*Chemicals, peptides, and recombinant proteins*		
TRIzol™ reagent	ThermoFisher Scientific	Cat# 15596026
Lipofectamine 3000 transfection reagent	ThermoFisher Scientific	Cat# L3000015
*Critical commercial assays*		
Cytofix/cytoperm fixation/ Permeabilization kit	BD Bioscience	Cat# 554714
cell staining buffer	Biolegend	Cat# 420201
*Experimental models: cell lines*		
Human: HEK293T	ATCC	Cat# CRL-3216; RRID: CVCL_0063
Human: U2OS	ATCC	Cat# SCSP-5030
Human: HCT116	ATCC	Cat# TCHu 99
Mouse: MC38	ATCC	Cat# HTX2138
Mouse: MEF	ATCC	Cat# CBP60599
*Experimental models: organisms/strains*		
C57BL/6 mice	Jackson laboratory	Cat# 000664
*Recombinant DNA*		
pCDH-mSENP3-WT	This paper	N/A
pCDH-mSENP3–9A	This paper	N/A
pCDH-SENP3-WT	This paper	N/A
pCDH-SENP3–9A	This paper	N/A
pCDH-SENP3–9E	This paper	N/A
pLVX-TetOne-Puro-SENP3-WT	This paper	N/A
pLVX-TetOne-Puro-SENP3–9A	This paper	N/A
Px459-mcGAS-Puro	This paper	N/A
Px459-SENP3-Puro	This paper	N/A
*Software and algorithms*		
FlowJo v.10.CL	FlowJo	RRID:SCR_008520
Prism v.7.0e	GraphPad	RRID:SCR_002798
*Other*		
BD FACSVerse^TM^	BD Biosciences	N/A

### Reporting summary

Further information on research design is available in the Nature Research Reporting Summary linked to this article.

## Supplementary information

SENP3-cGAS supple 2022-06-23

SENP3-cGAS supple -western blot

Reporting Summary

## Data Availability

Any additional information required to reanalyze the data reported in this paper is available from the lead contact upon request.
