# Supplementary material for: Mitotic SENP3 activation couples with cGAS signaling in tumor cells to stimulate anti-tumor immunity
Source: Cell Death Dis. 2022 Jul 22;13(7):640. doi: 10.1038/s41419-022-05063-6 (PMC9307842; doi:10.1038/s41419-022-05063-6)
Supplement: Supplementary file 1 — SENP3-cGAS supple 2022-06-23 [file 41419_2022_5063_MOESM1_ESM.docx]

**Supplemental Data**

**Mitotic SENP3 activation couples with cGAS signaling in tumor cells to stimulate anti-tumor immunity**

Gaolei Hu, Yalan Chen, Xinyu Yang, Yang Wang, Jianli He, Tianshi Wang, Qiuju Fan, Liufu Dong, Jun Tu, Hongsheng Tan, and Jinke Cheng


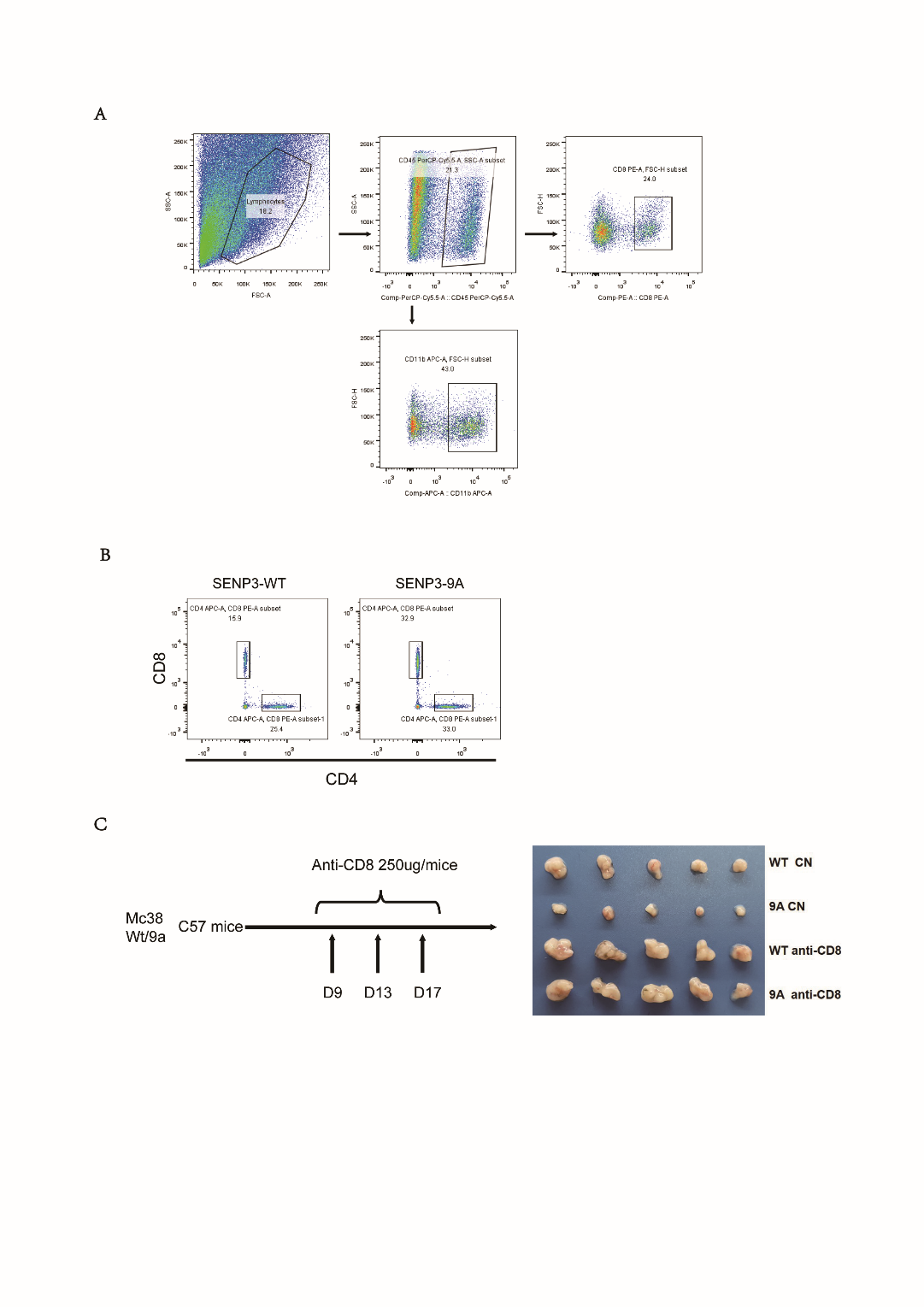


**Figure S1.** Activating mitotic SENP3 in tumor cells stimulates host anti-tumor immunity.

(**A**) SENP3-WT-MC38 cells (WT) or SENP3-9A-MC38(9A) cells were subcutaneously injected into C57BL/6 mice (1x106/mouse, n = 5 mice). Tumors were harvested in 18 days’ post-injection and analyzed by flow cytometry. The cells were pre-gated on CD45^+^.

(**B**) SENP3-WT-MC38 cells (WT) or SENP3-9A-MC38(9A) cells were subcutaneously injected into C57BL/6 mice (1x10^6^/mouse, n = 5 mice). Tumor draining inguinal lymph nodes (dLNs) were removed from mice in 18 days’ post-injection. CD8^+^ or CD4^+^ T cells in CD45^+^ cells were analyzed by flow cytometry.

(**C**) GFP-tagged SENP3-WT-MC38 cells (WT) or GFP-tagged SENP3-9A-MC38(9A) cells were subcutaneously injected into C57BL/6 mice (1x10^6^/mouse, n = 5 mice). On day 7 after injection, each kind of tumor-bearing mice were divided into two groups and anti-mouse CD8α antibody were intraperitoneally injected (250μg per mouse) on day 9, 13, and 17 after tumor injection. Tumor size was determined once in 3 days in two dimensions.


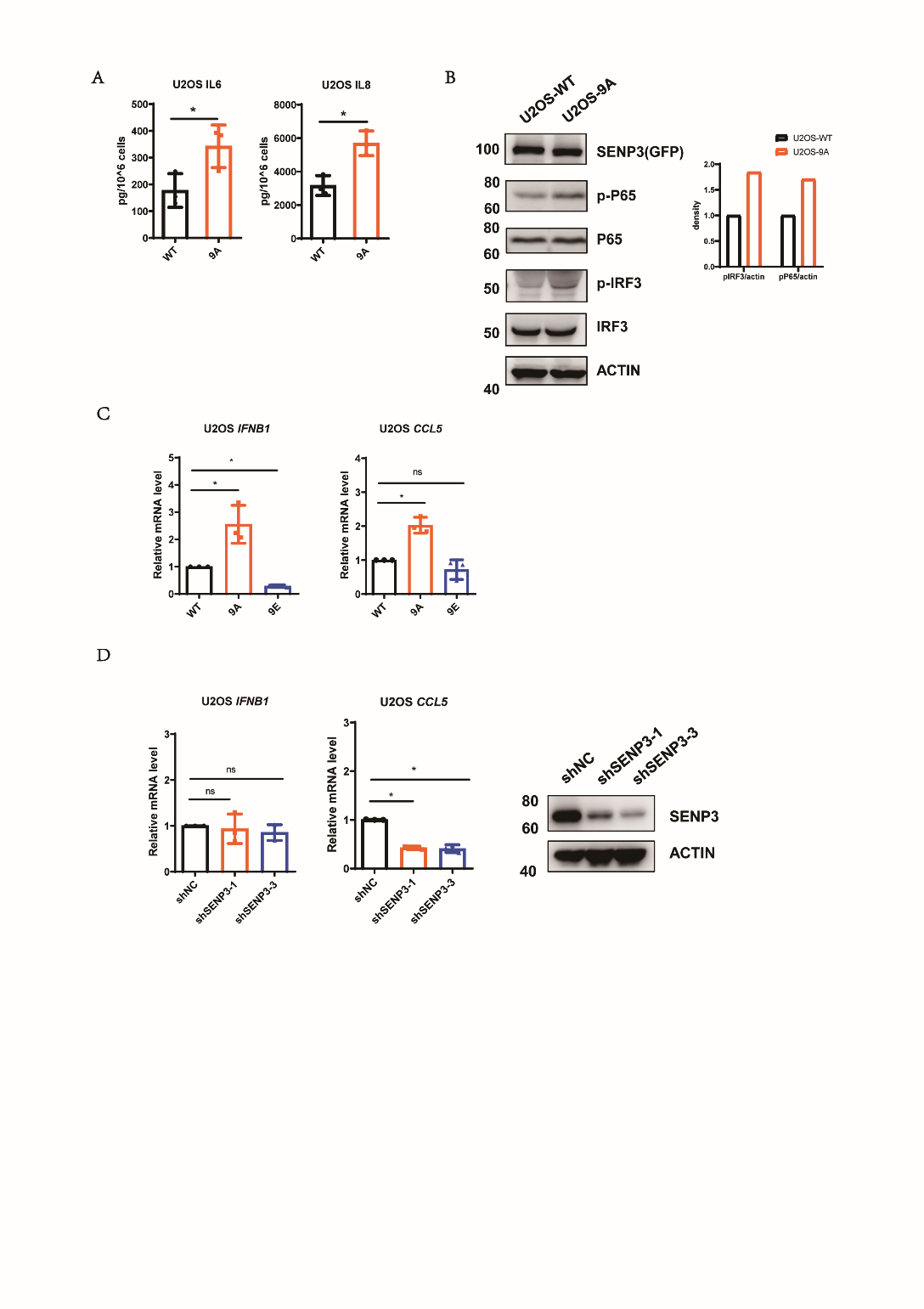


**Figure S2**. Mitotic SENP3 activates cGAS signaling.

(**A**) U2OS cell lines were stably transfected with GFP-tagged human SENP3-WT or SENP3-9A, the concentration of IL6 and IL8 in culture medium was detected by ELISA in 6 days after transfection. Data are represented as mean with SD. (_*_) P<0.05

(**B**) U2OS cell lines were stably transfected with GFP-tagged human SENP3-WT or SENP3-9A, and phosphorylation of IRF3 and p65 were analyzed by immunoblotting in 6 days after transfection.

(**C**) U2OS cell lines were stably transfected with GFP-tagged human SENP3-WT, SENP3-9A or SENP3-9E. Real-time PCR was used for analysis on *IFNB1, CCL5* expression in these cells. The data were normalized to GAPDH internal control and are representative of three independent experiments. Data are represented as mean with SD. (_*_) P<0.05

(D) U2OS cell lines were stably transfected with non-targeting control (shNC) or sh-SENP3 and analyzed by immunoblotting. Real-time PCR was used for analysis on *IFNB1, CCL5* expression in these cells. The data were normalized to GAPDH internal control and are representative of three independent experiments. Data are represented as mean with SD. (_*_) P<0.05


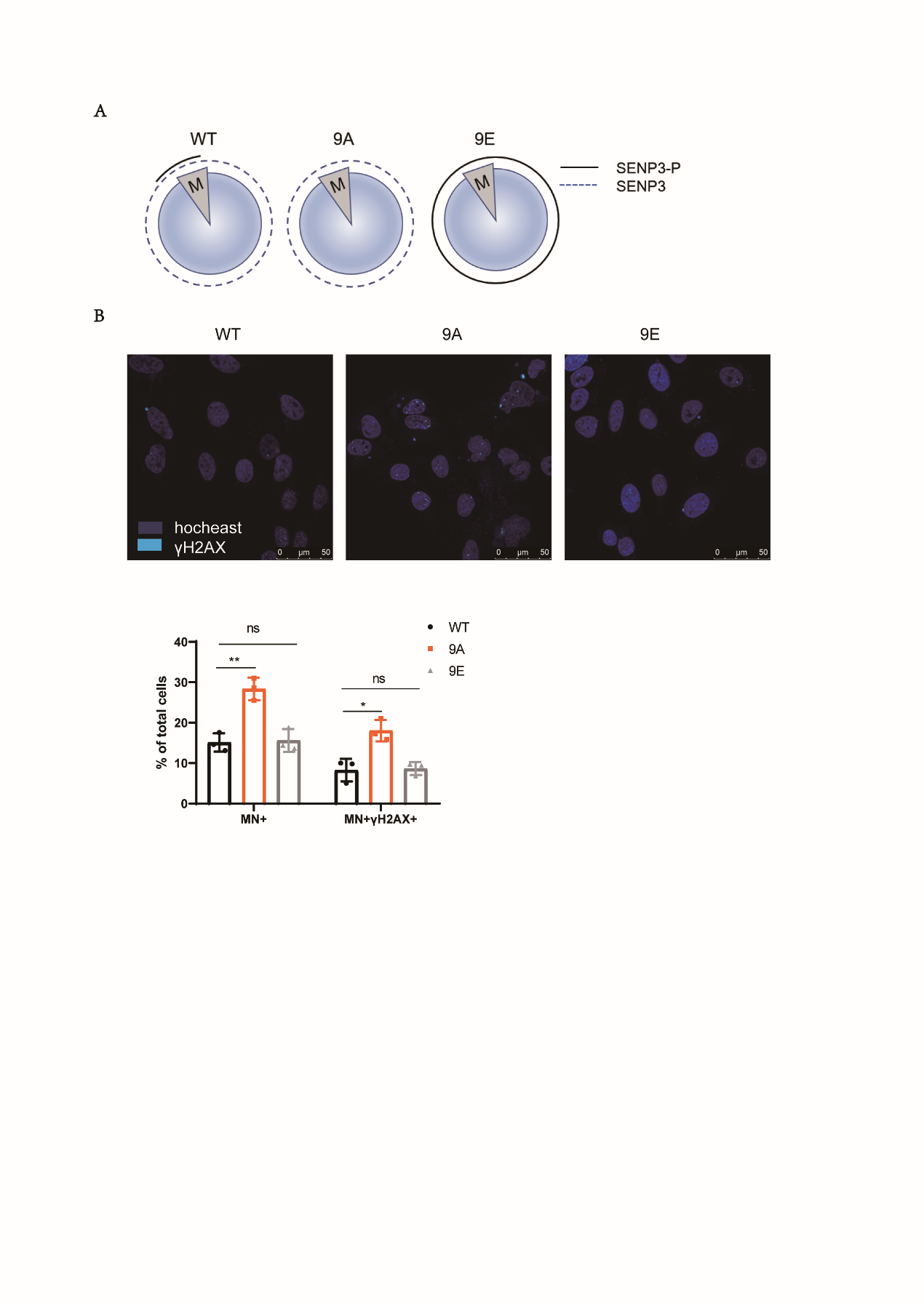


**Figure S3**. Mitotic SENP3 activation promotes micronucleus formation.

(**A, B**) U2OS cell lines stably transfected with human SENP3-WT, SENP3-9A or SENP3-9E were stained with Hoechst one week after transfection. The micronucleus staining positive cells (percentage of total cells) were quantitatively analyzed on 30 fields pictured by confocal microscope. Data are represented as mean with SD. (_**_) P<0.01. (_*_) P<0.05


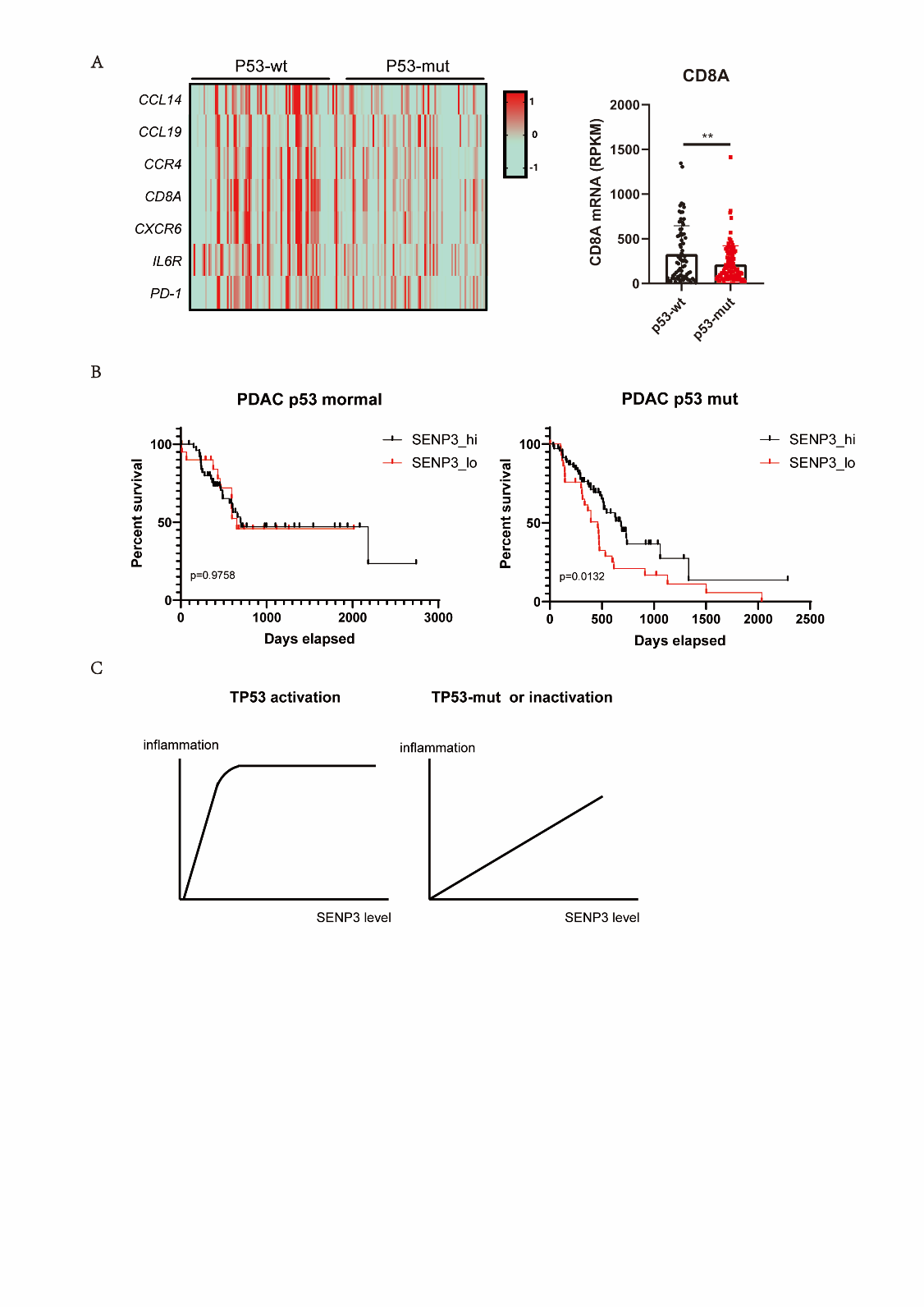


**Figure S4**. SENP3 expression positively correlated with immune response in p53 mutant pancreatic cancer patients^40^.

(**A**) Heatmaps show that immune response related genes are mildly higher in p53 activation group than in p53 mutant group.

(**B**) Pancreatic cancer patients were divided according to p53 status (p53-WT or p53 mutant). SENP3 expression level positively correlate with better survival of human pancreatic cancer patients in p53 mutant group but not in p53-WT group. Groups of patients with high expression levels of SENP3 were compared with those with low expression levels and Kaplan–Meier curves were calculated.

(**C**) Proposed model for the Influence of p53 status on correlation between SENP3 expression level and inflammation response. Mitotic SENP3 could be fully activated through p53 signaling induced by DNA damage and the amount of mitotic activated SENP3 could maintain at a high-level regard less of SENP3 expression level. However, when p53 is mutated or inactivated, then only a small portion of SENP3 could be activated during mitosis and promote inflammation response, then the amount of mitotic activated SENP3 may be positively corelated with SENP3 expression level.

**Supplementary Table1. qPCR primers for innate immunity related genes**

| Primers | Sequence |
| --- | --- |
| *mCcl5-F* | TTTGCCTACCTCTCCCTCG |
| *mCcl5-R* | CGACTGCAAGATTGGAGCACT |
| *mIsg15-F* | GGTGTCCGTGACTAACTCCAT |
| *mIsg15-R* | CTGTACCACTAGCATCACTGTG |
| *mIfnb1-F* | AGCTCCAAGAAAGGACGAACA |
| *mIfnb1-R* | GCCCTGTAGGTGAGGTTGAT |
| *mCxcl10-F* | CCAAGTGCTGCCGTCATTTTC |
| *mCxcl10-R* | GGCTCGCAGGGATGATTTCAA |
| *mIl6-F* | CTGCAAGAGACTTCCATCCAG |
| *mIl6-R* | AGTGGTATAGACAGGTCTGTTGG |
| *mGapdh-F* | GTCATCCCAGAGCTGAACG |
| *mGapdh-R* | TCATACTTGGCAGGTTTCTCC |
| *Hs IL6-F* | ACTCACCTCTTCAGAACGAATTG |
| *Hs IL6-R* | CCATCTTTGGAAGGTTCAGGTTG |
| *Hs CCL5-F* | CCAGCAGTCGTCTTTGTCAC |
| *Hs CCL5-R* | CTCTGGGTTGGCACACACTT |
| *Hs IFNB1-F* | GCTTGGATTCCTACAAAGAAGCA |
| *Hs IFNB1-\|R* | ATAGATGGTCAATGCGGCGTC |
| *Hs GAPDH-F* | GTCTCCTCTGACTTCAACAGCG |
| *Hs GAPDH-R* | ACCACCCTGTTGCTGTAGCCAA |
