# Supplementary material for: Mitotic SENP3 activation couples with cGAS signaling in tumor cells to stimulate anti-tumor immunity
Source: Cell Death Dis. 2022 Jul 22;13(7):640. doi: 10.1038/s41419-022-05063-6 (PMC9307842; doi:10.1038/s41419-022-05063-6)

**Original western blot figure:**

**Fig 2B**

GFP-SENP3


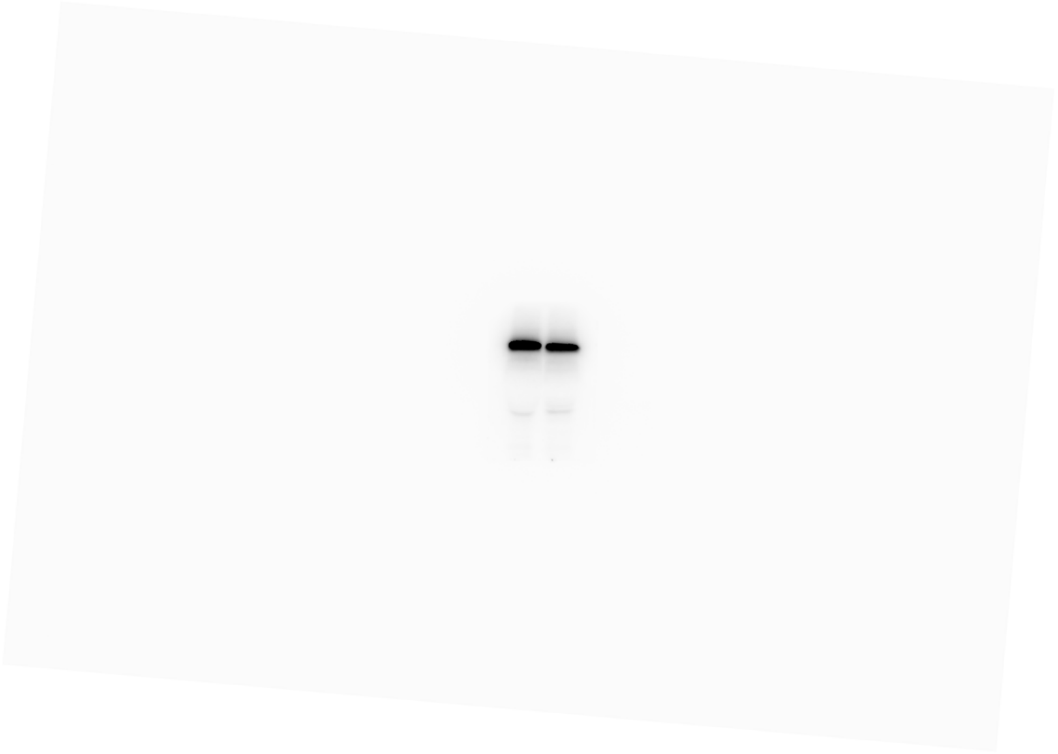


p-TBK1 line 2-3


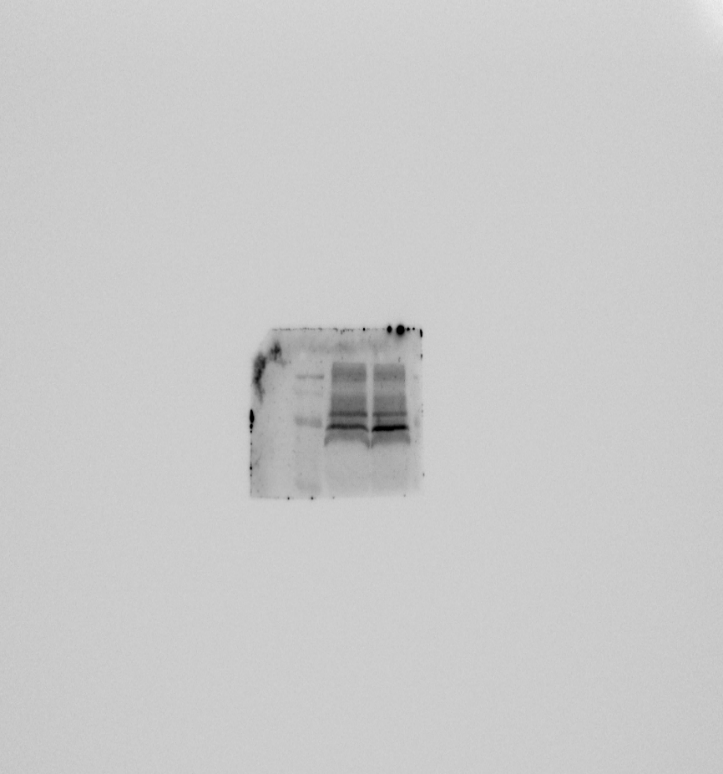

TBK1 line 4-5


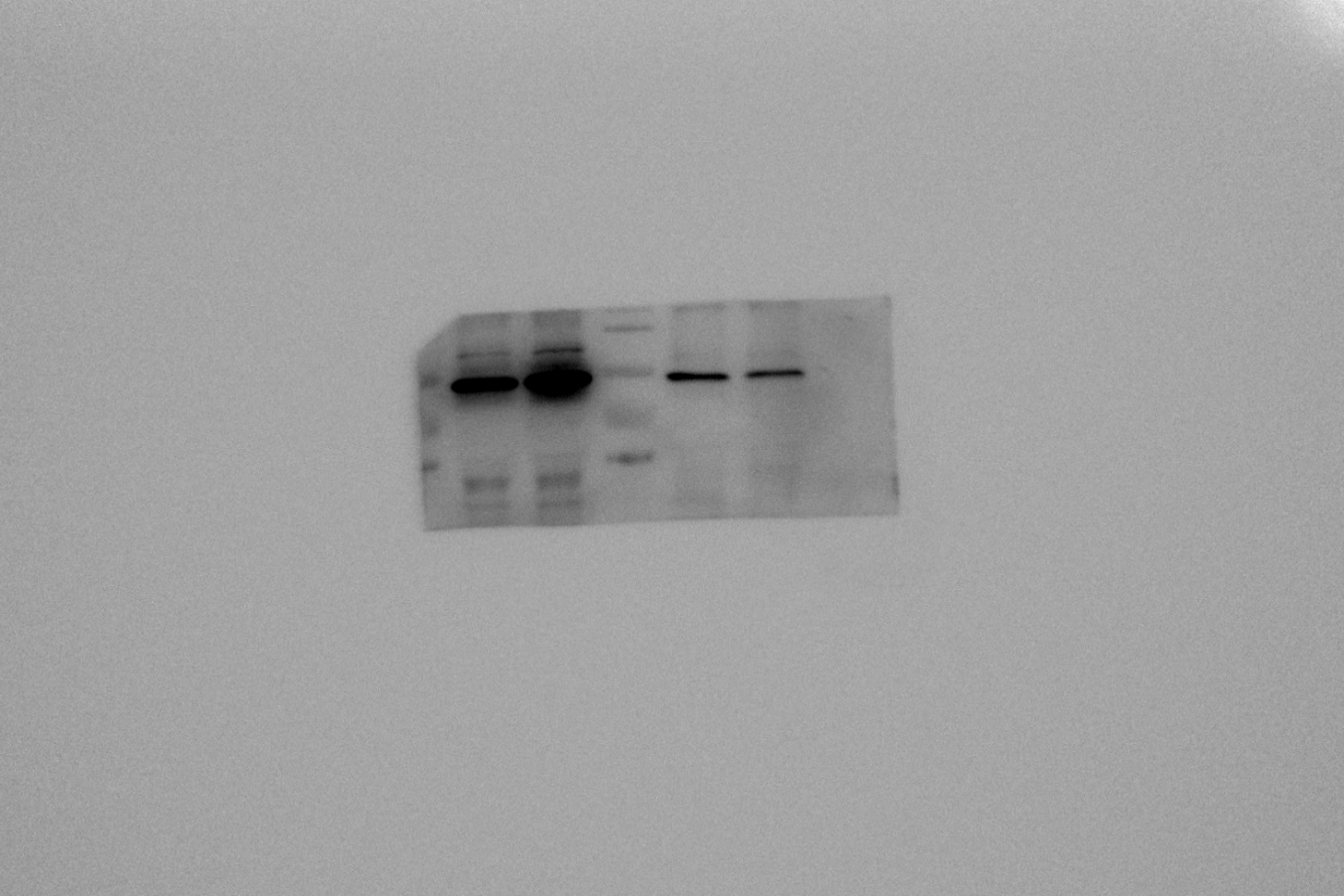


pP65


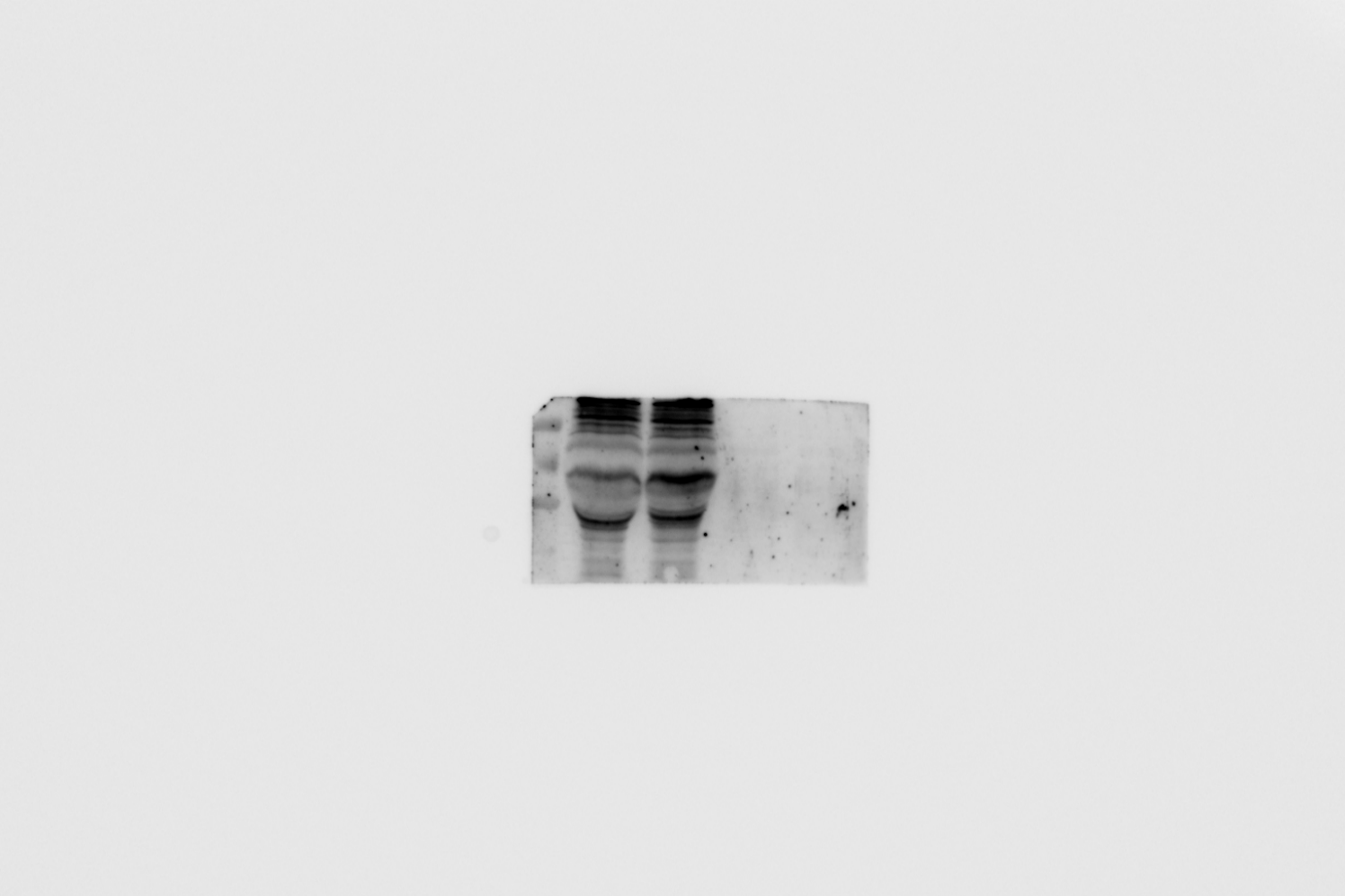


P65 line 5-6


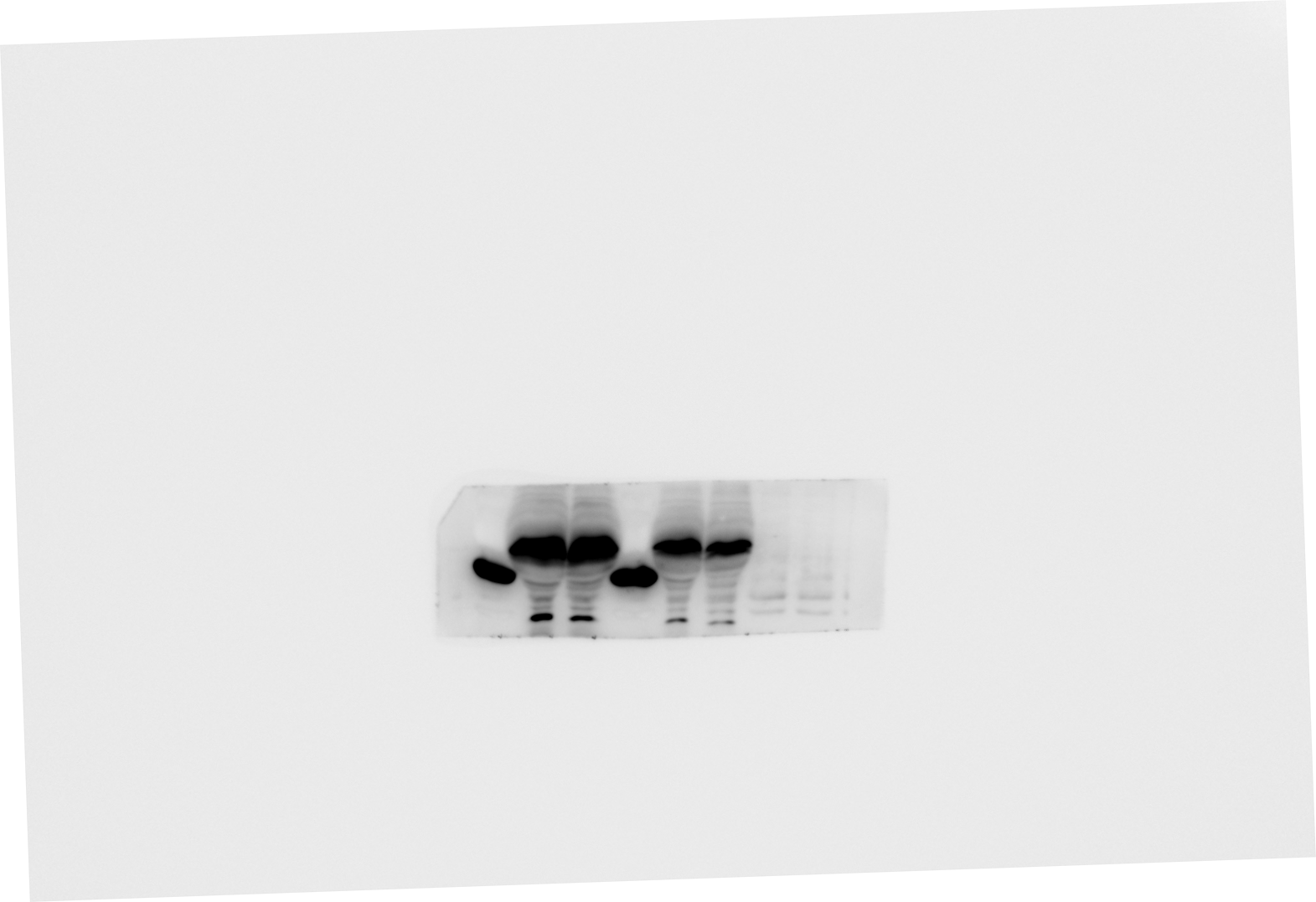


actin


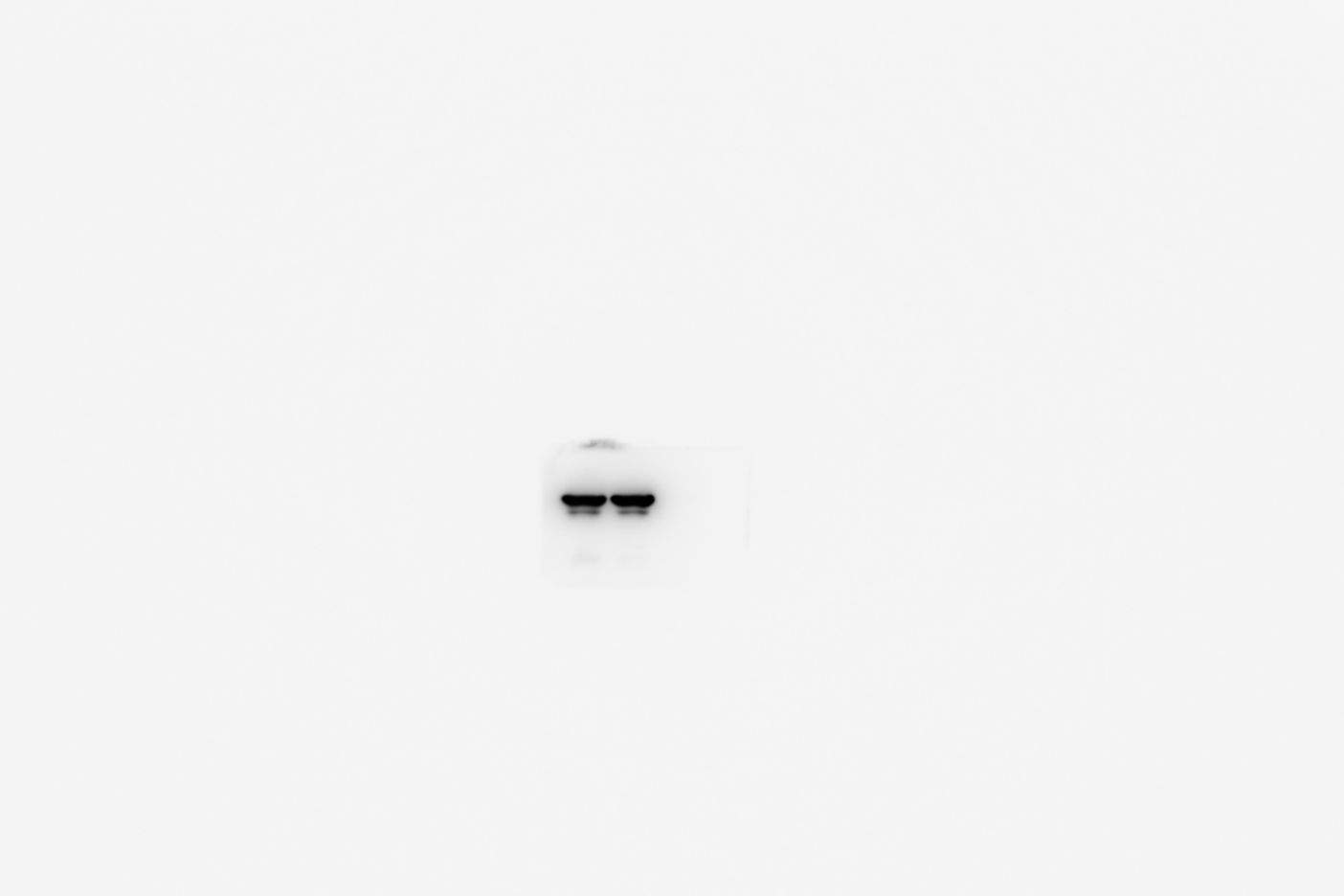


**Fig 2D**

**cGAS** line 2-3

**
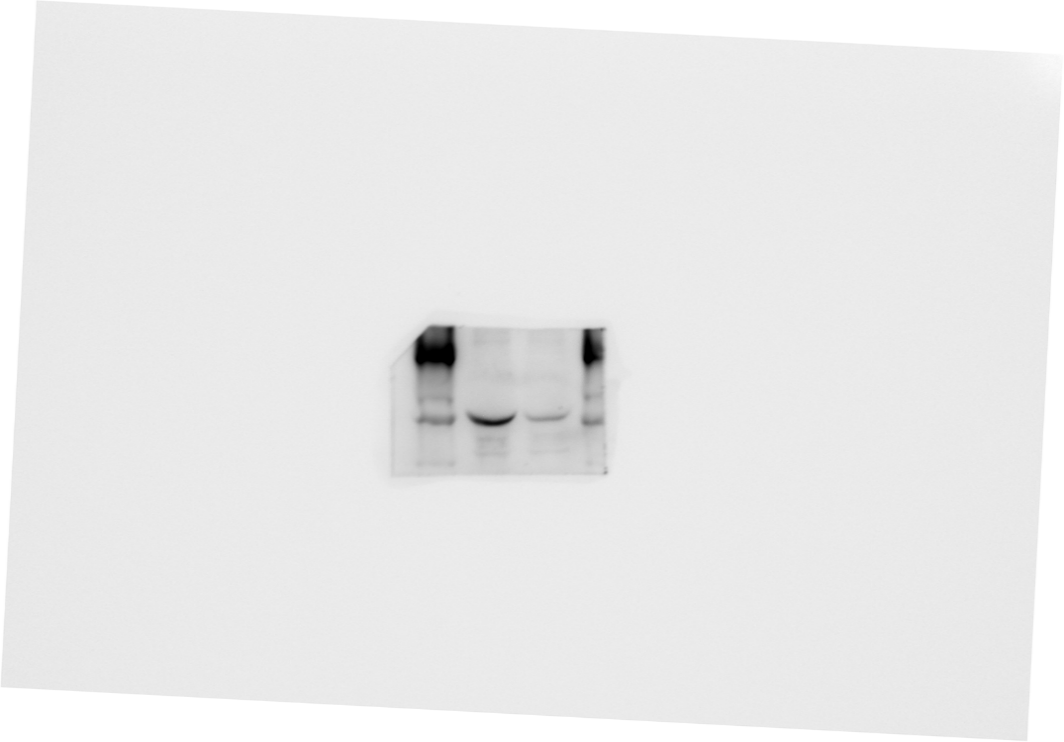
**

**Actin**

**
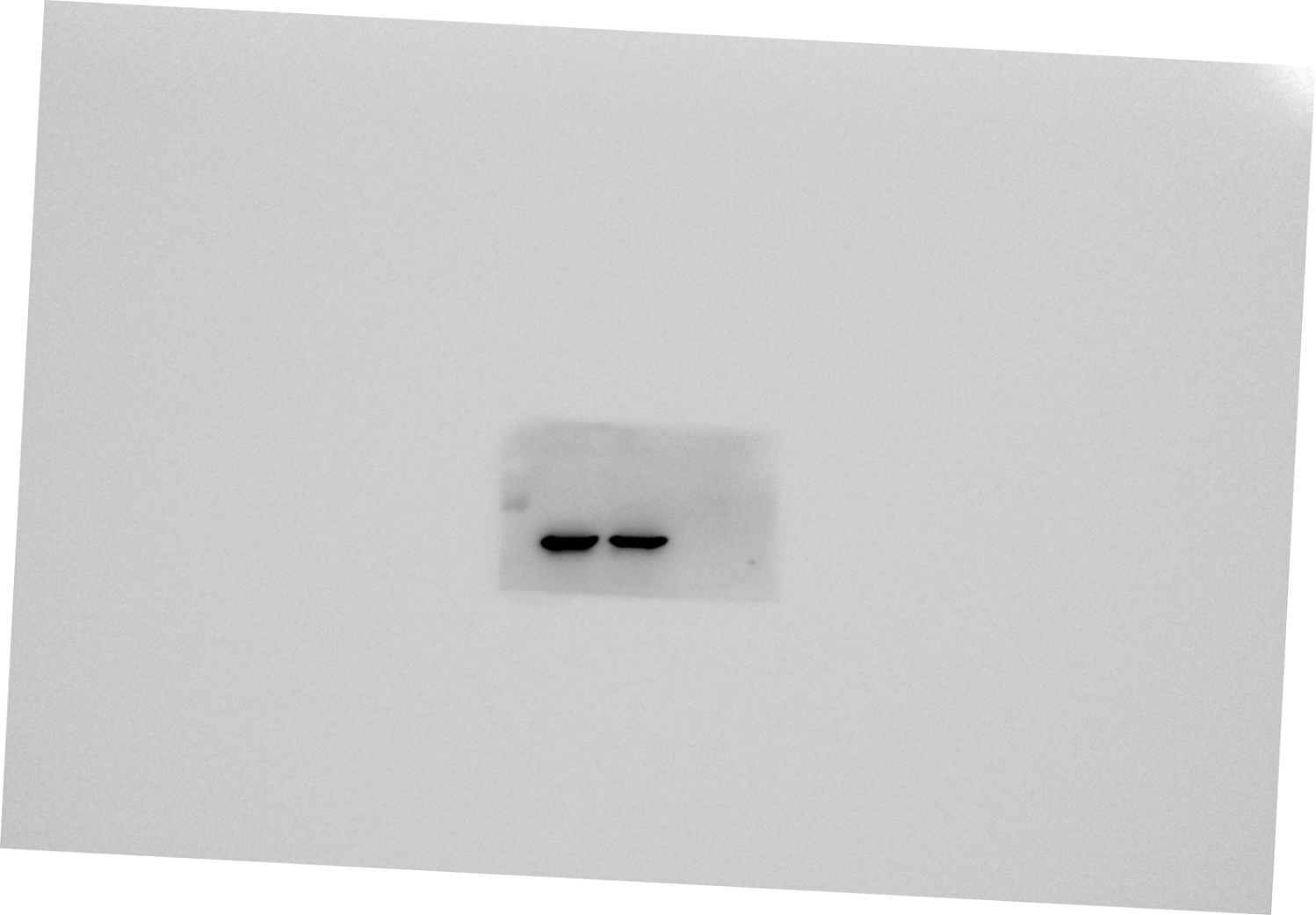
**

**Fig 3B**

SENP3

**
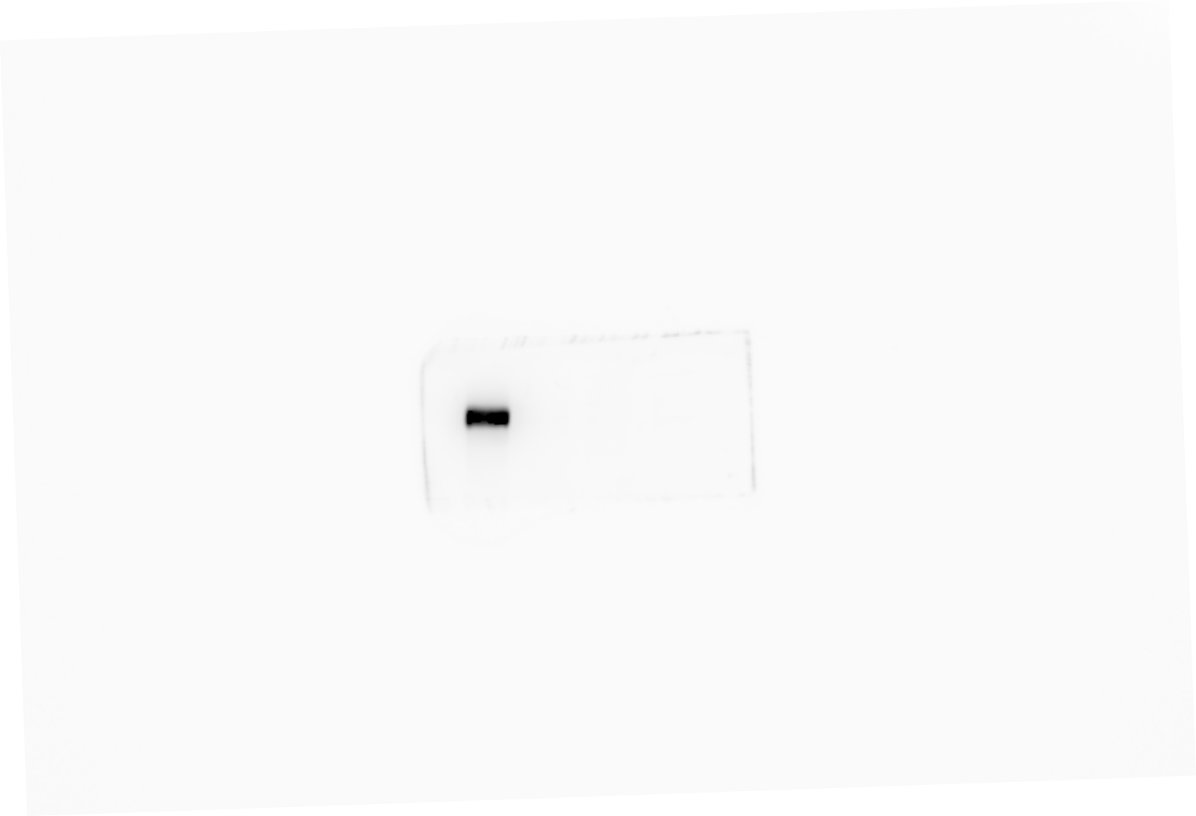
**

**Actin line1-2**

**
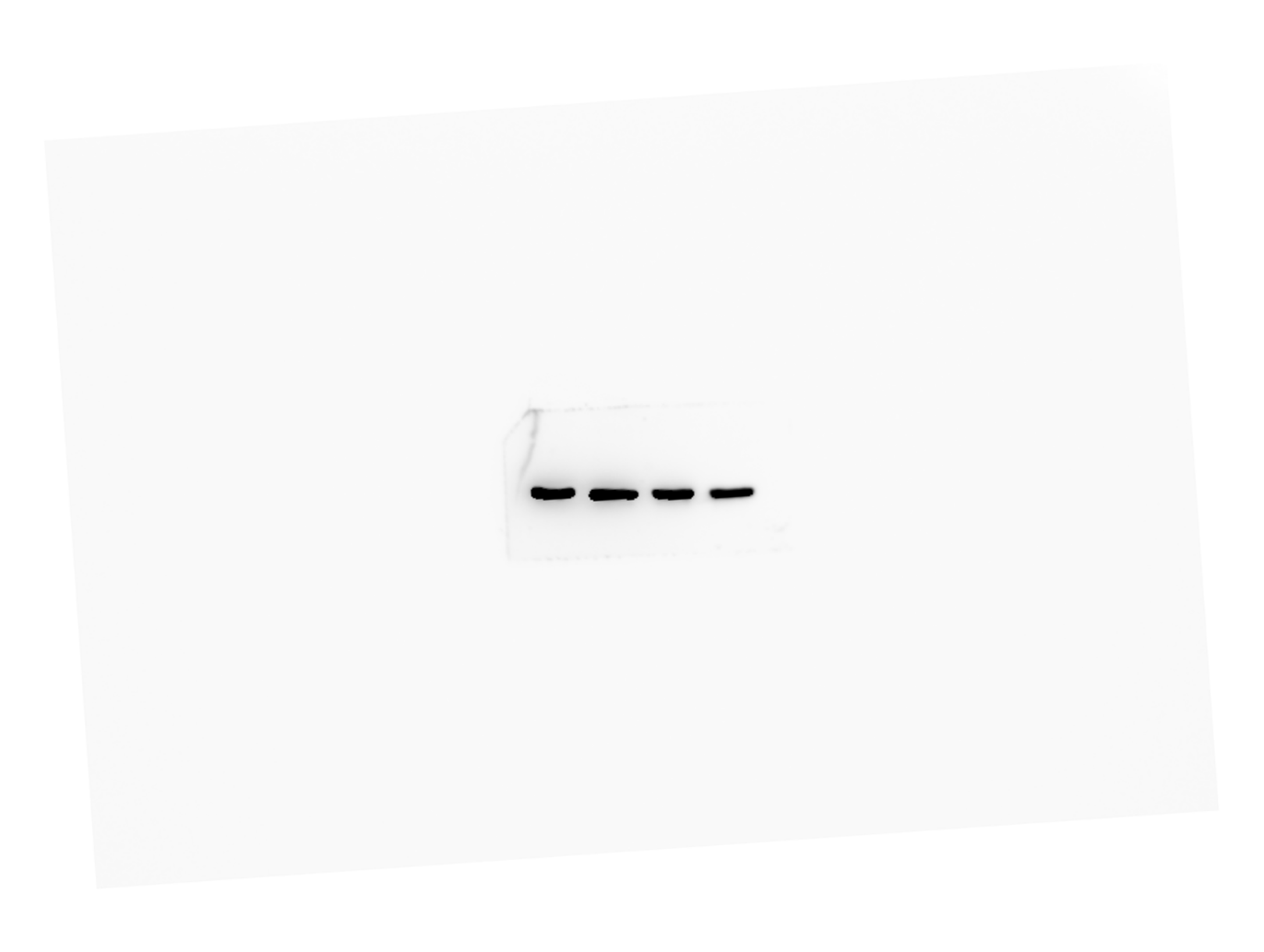
**

**SENP3**

**
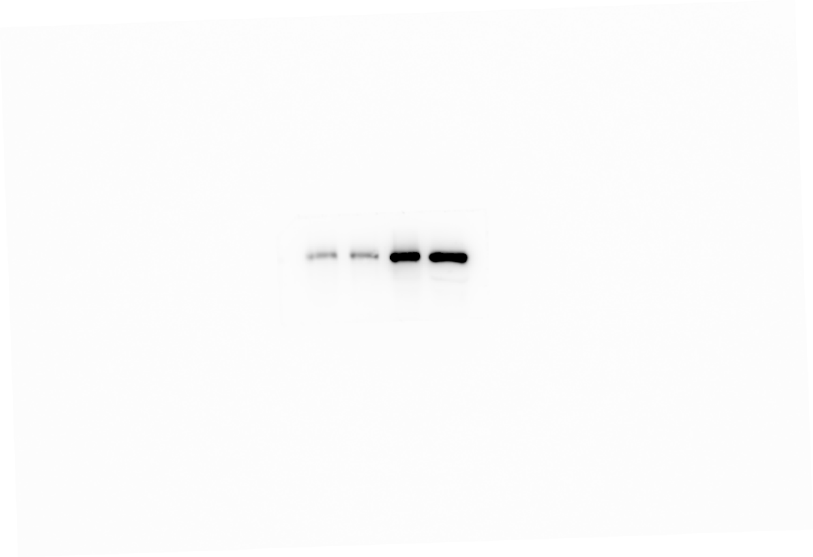
**

**Actin**

**
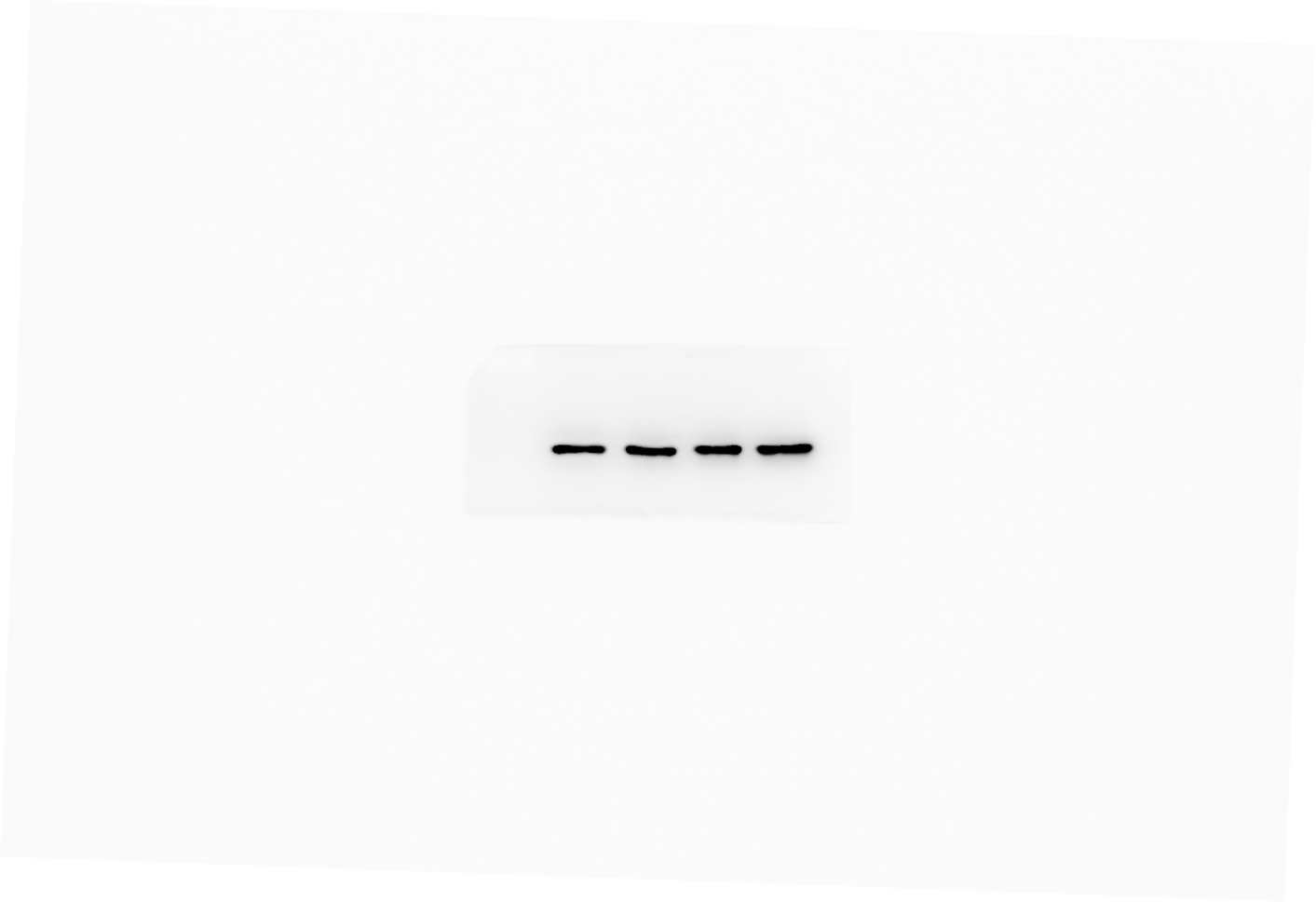
**

**Fig S2B**

**GFP-SENP3**

**
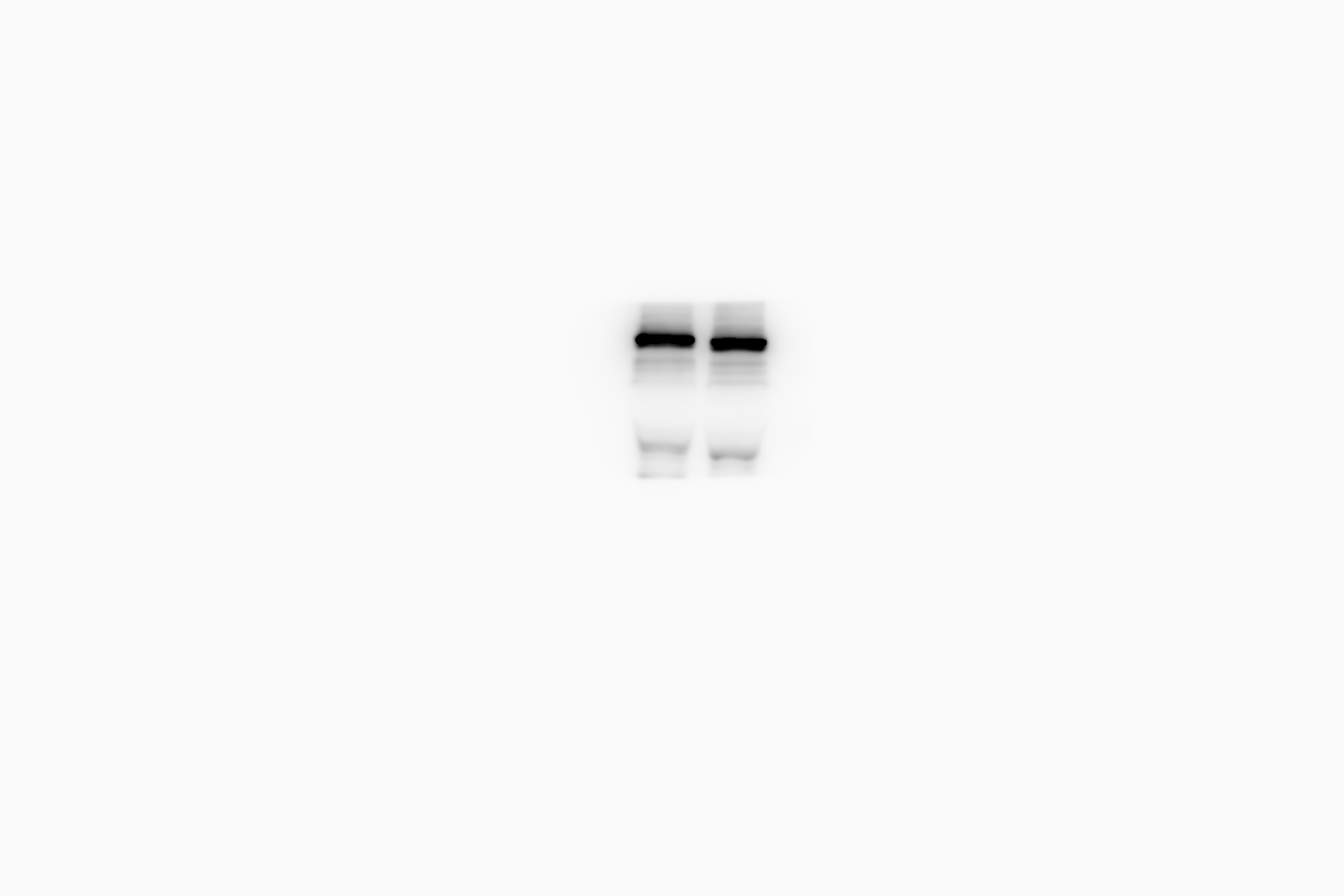
**

**P-P65** line 1-2

**
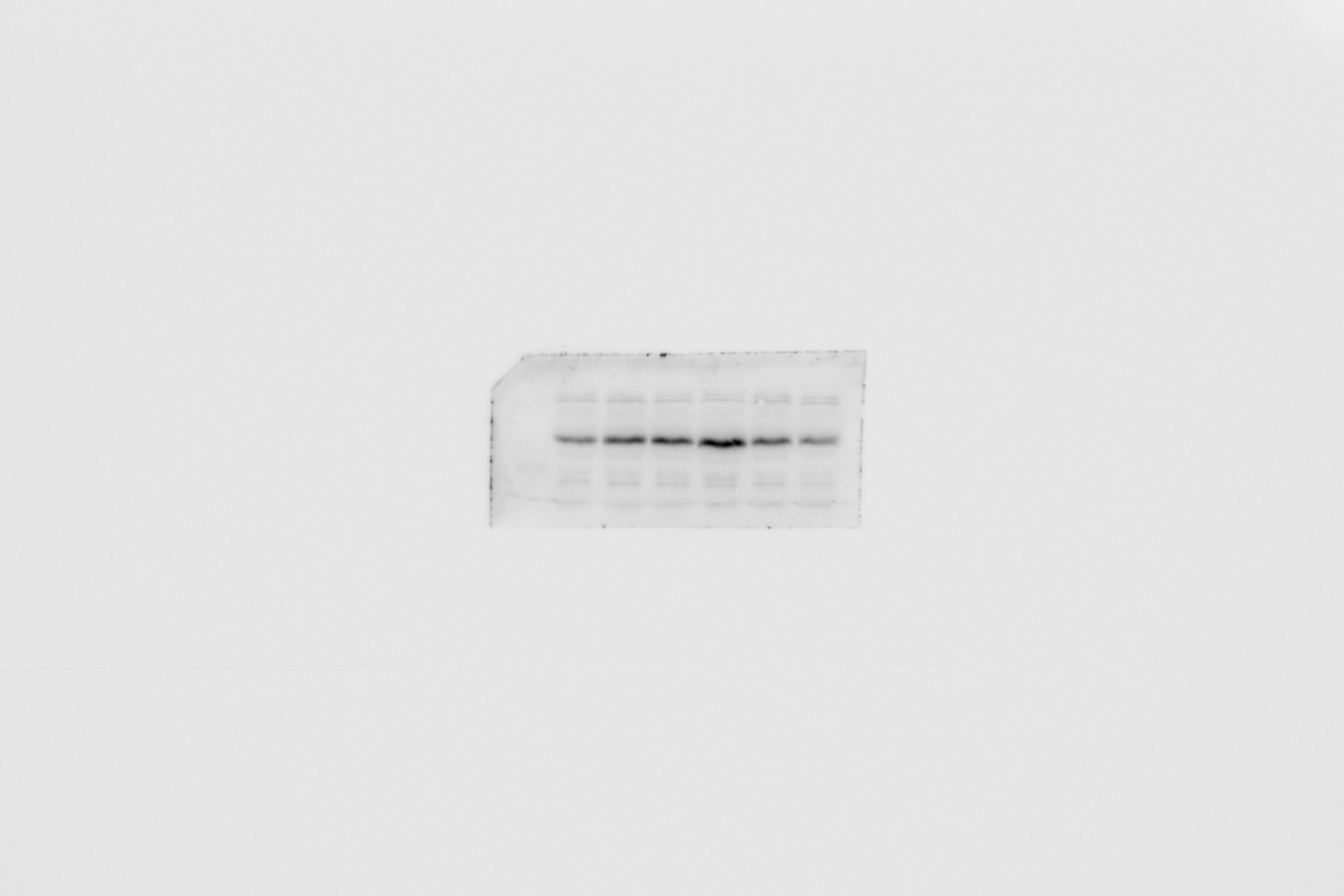
**

**P65** line 1-2

**
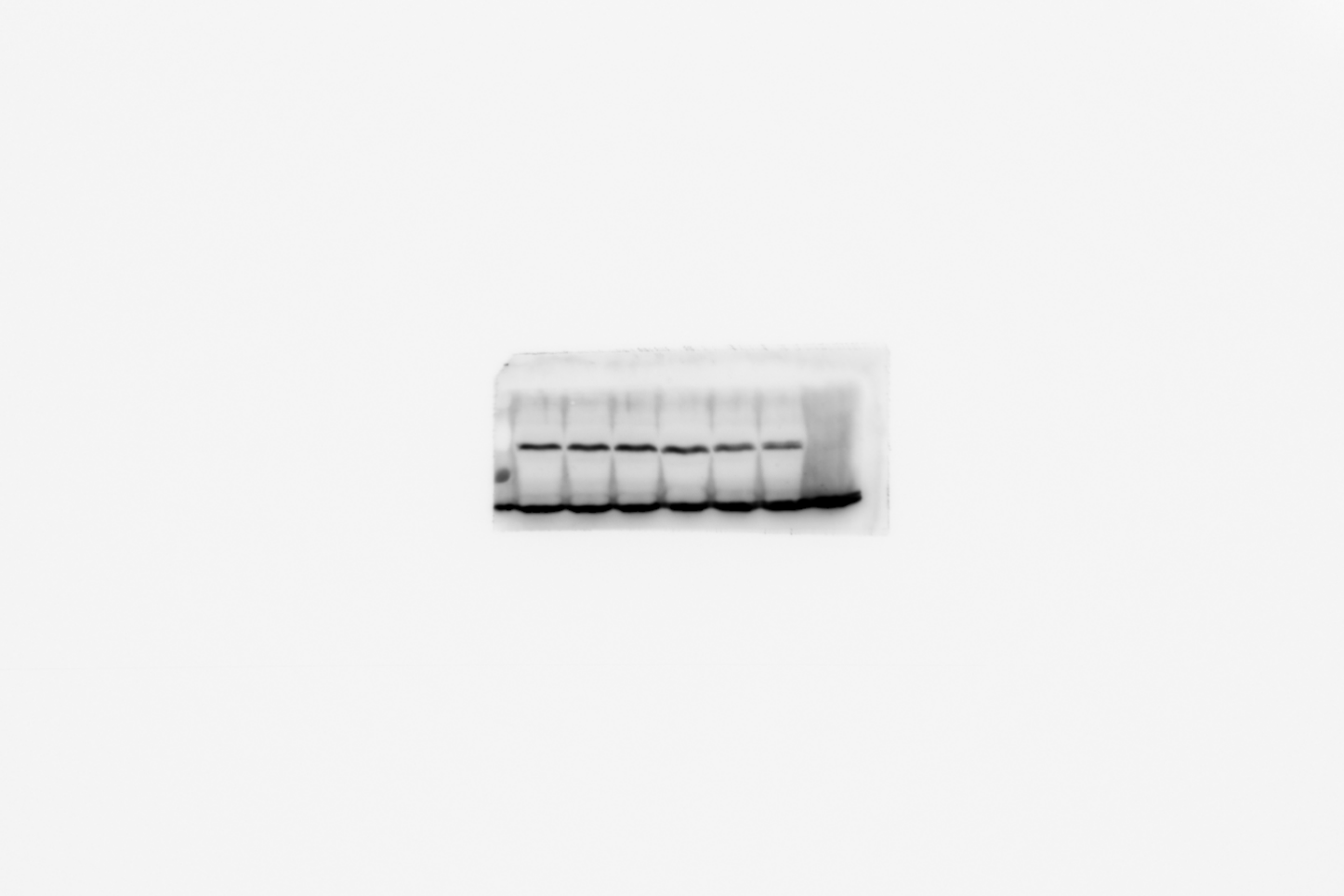
**

**P-IRF3** line 2-3

**
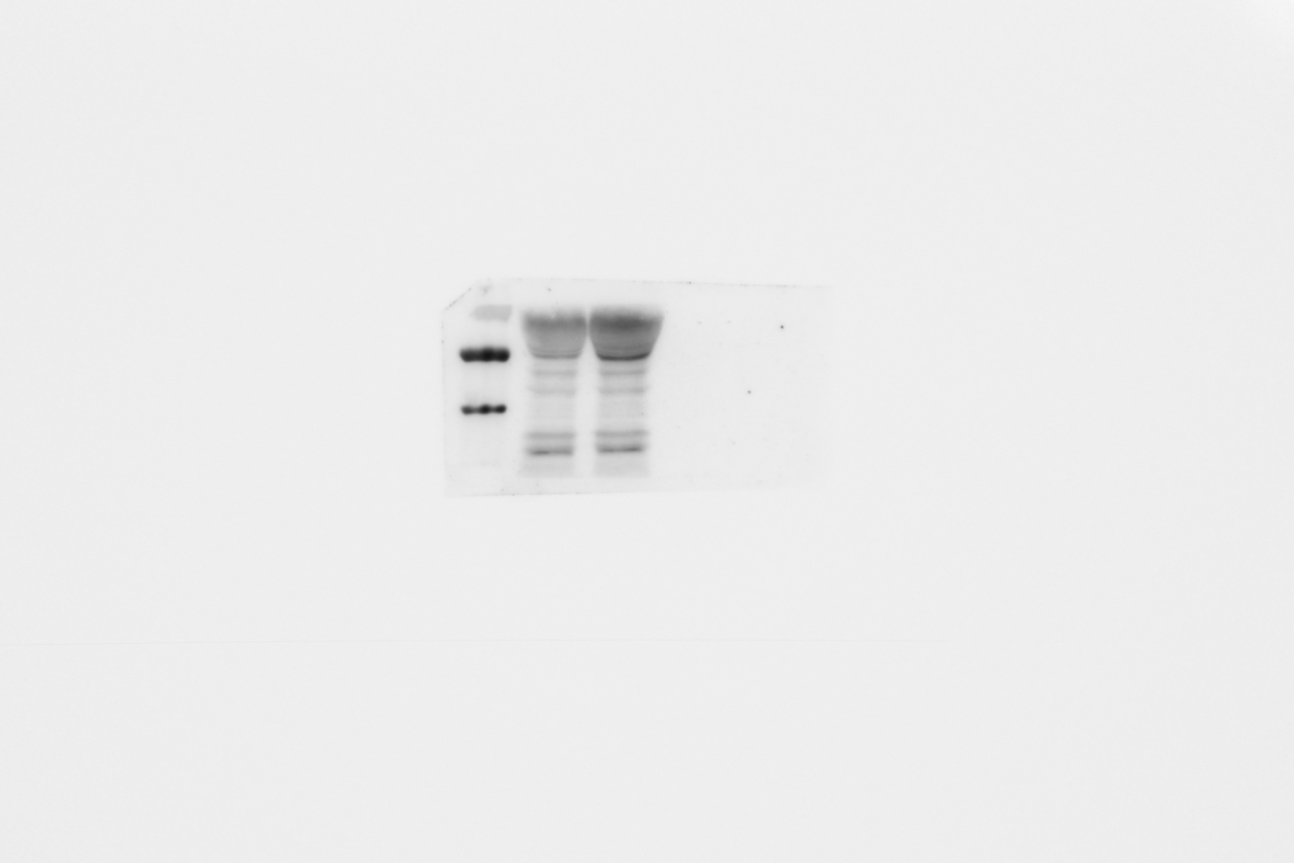
**

**IRF3** line 2-3

**
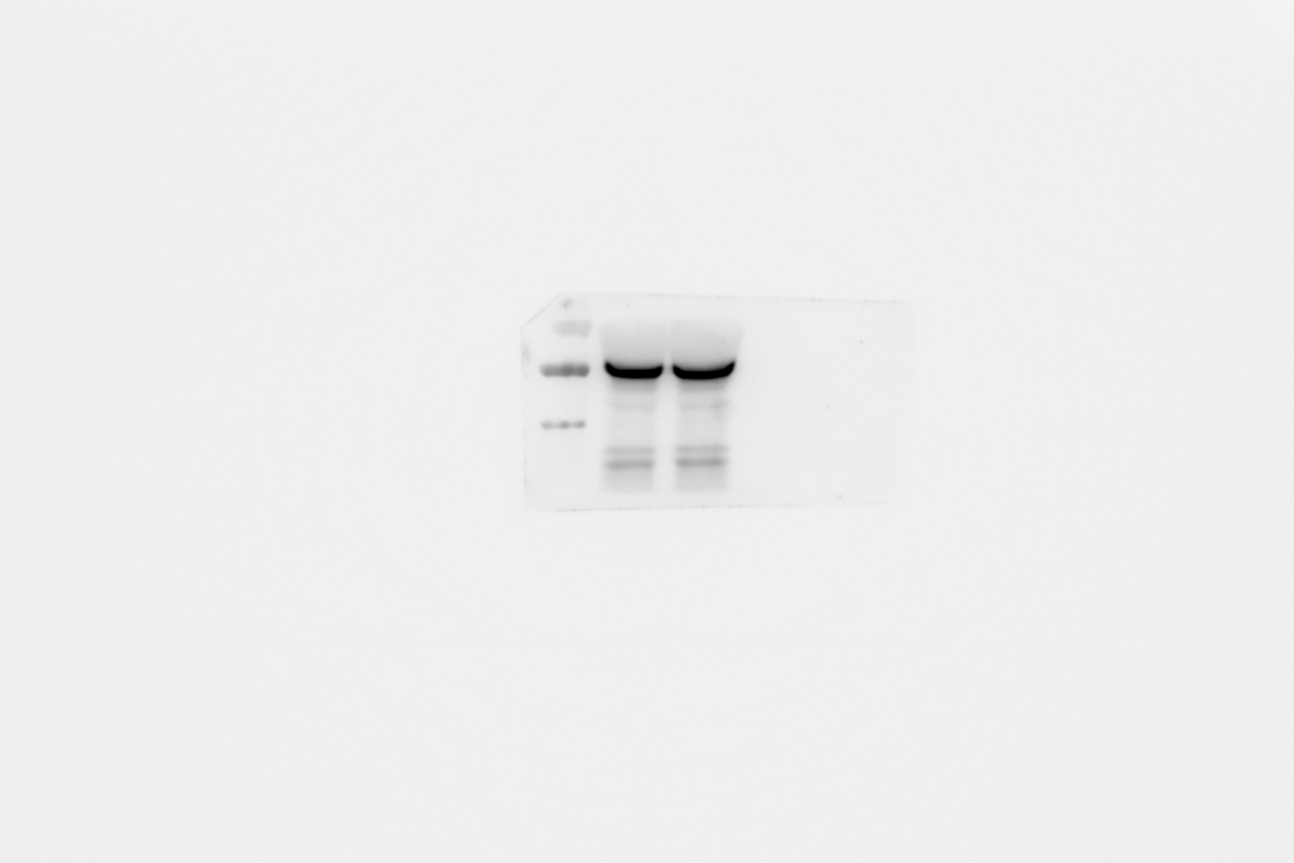
**

**Actin** line 1-2

**
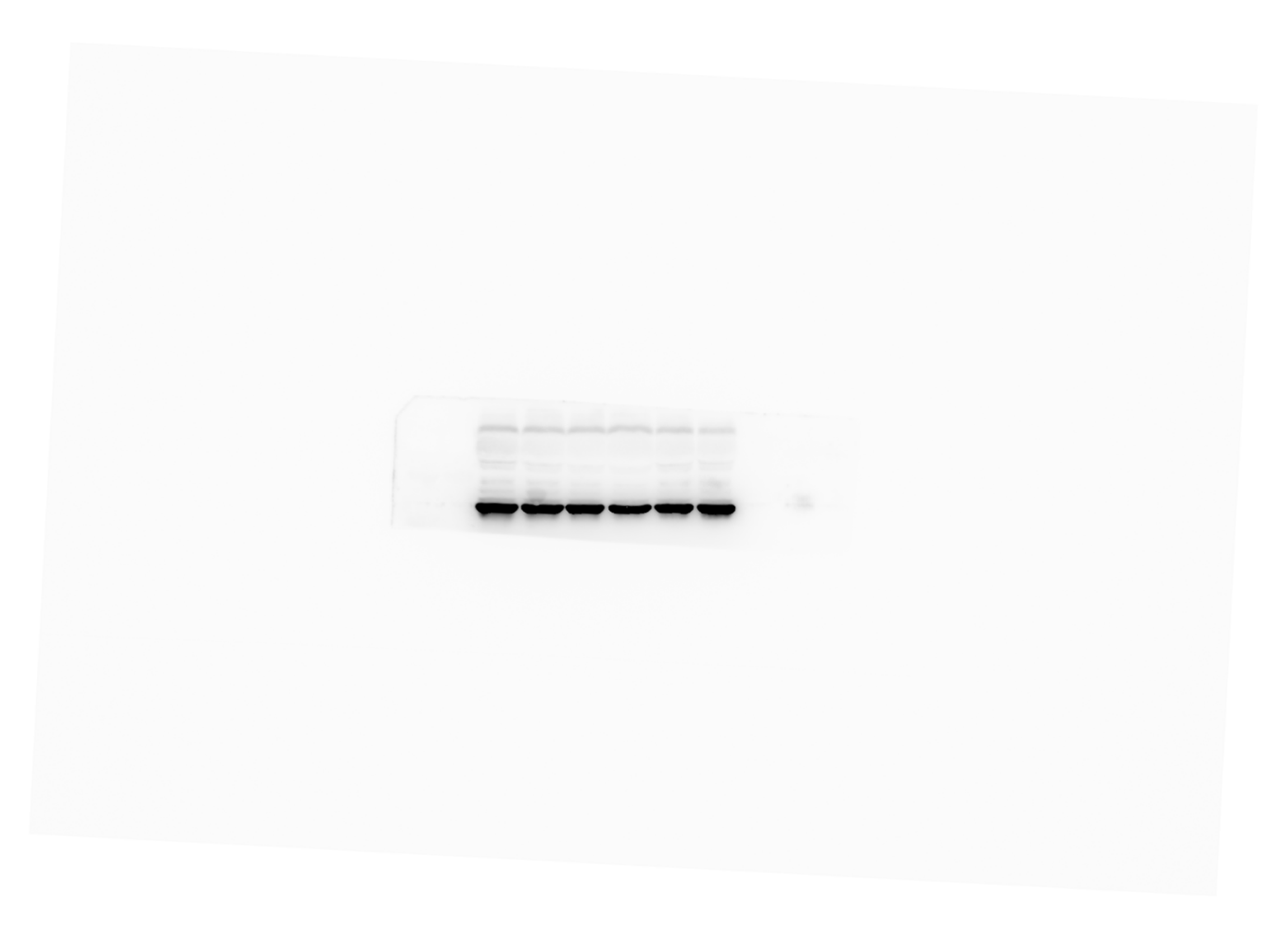
**

**Fig S2D**

**SENP3**


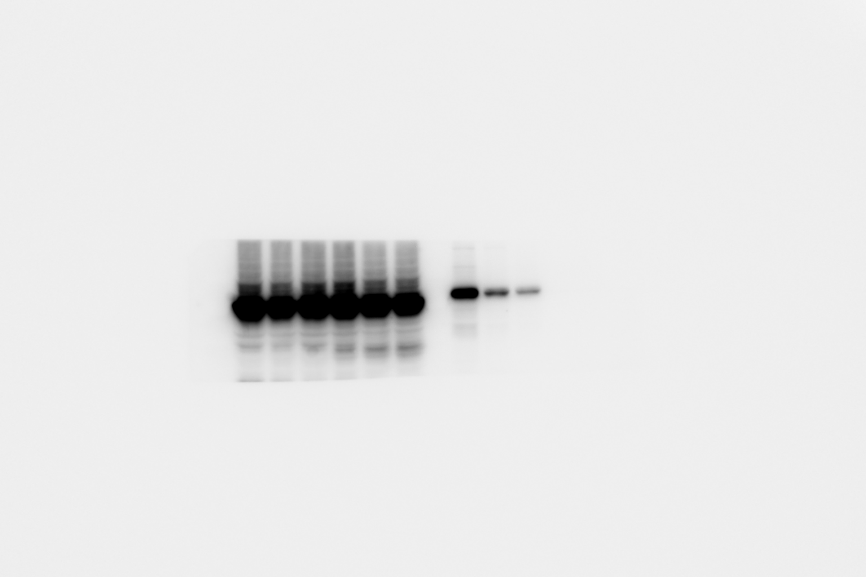


**ACTIN**


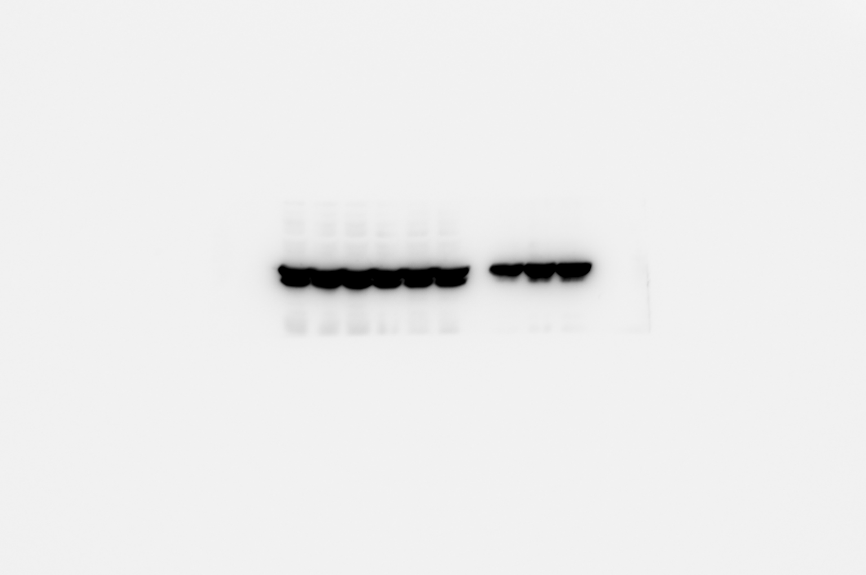

Supplement: Supplementary file 2 — SENP3-cGAS supple -western blot [file 41419_2022_5063_MOESM2_ESM.docx]
